# Nucleic Acid-Binding Dyes as Versatile Photocatalysts for Atom-Transfer Radical Polymerization

**DOI:** 10.1021/jacs.4c03513

**Published:** 2024-05-01

**Authors:** Jaepil Jeong, Xiaolei Hu, Rongguan Yin, Marco Fantin, Subha R. Das, Krzysztof Matyjaszewski

**Affiliations:** † Department of Chemistry, Carnegie Mellon University, Pittsburgh, Pennsylvania 15213, United States; ‡ Center for Nucleic Acids Science & Technology, Carnegie Mellon University, Pittsburgh, Pennsylvania 15213, United States; § Department of Chemical Sciences, University of Padova, Via Marzolo 1, Padova 35131, Italy

## Abstract

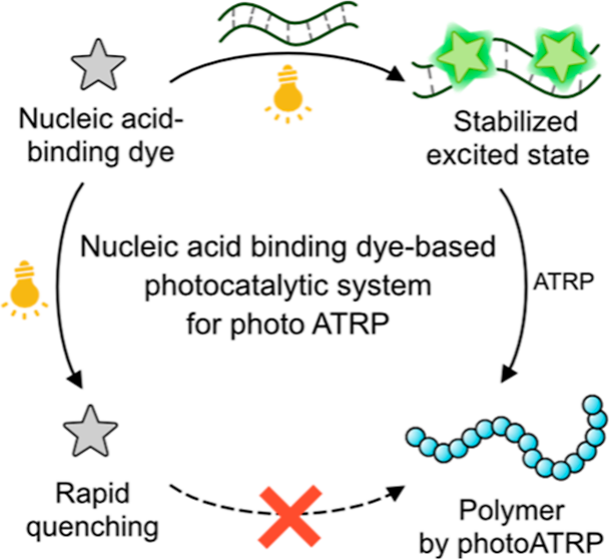

Nucleic acid-binding dyes (**NuABDs**) are fluorogenic probes that light up after binding to nucleic acids. Taking advantage of their fluorogenicity, **NuABDs** have been widely utilized in the fields of nanotechnology and biotechnology for diagnostic and analytical applications. We demonstrate the potential of **NuABDs** together with an appropriate nucleic acid scaffold as an intriguing photocatalyst for precisely controlled atom-transfer radical polymerization (ATRP). Additionally, we systematically investigated the thermodynamic and electrochemical properties of the dyes, providing insights into the mechanism that drives the photopolymerization. The versatility of the **NuABD**-based platform was also demonstrated through successful polymerizations using several **NuABDs** in conjunction with diverse nucleic acid scaffolds, such as G-quadruplex DNA or DNA nanoflowers. This study not only extends the horizons of controlled photopolymerization but also broadens opportunities for nucleic acid-based materials and technologies, including nucleic acid–polymer biohybrids and stimuli-responsive ATRP platforms.

## Introduction

Reversible deactivation radical polymerization (RDRP) techniques are changing the world by enabling the controlled synthesis of polymers with desired properties.^1−4^ Throughout the RDRP processes, propagating chains undergo a reversible activation/deactivation process. This is achieved by transition-metal complexes (typically, Cu in atom-transfer radical polymerization, ATRP) or chain-transfer agents (CTAs) (typically, thiocarbonylthio compounds, in reversible addition–fragmentation chain-transfer, RAFT).^5,6^ The RDRP-regulating reagents can be controlled by using external stimuli. Among them, light-mediated RDRP has gained broad interest due to its convenient spatiotemporal control. Under light irradiation, photocatalysts undergo excitation, followed by activation of RDRP-regulating reagents via electron/energy transfer or the direct generation of propagating radicals.^7,8^ Over the past few decades, various photosensitizers, from chromophores and photoinitiators to metal-based compounds, have been explored as mediators of RDRP processes.^9^

In addition to simple small molecules, multidimensional photocatalysts and new photocatalytic platforms have also been tested, opening up new opportunities.^10^ For instance, the development of heterogeneous photocatalysts, through the immobilization of photosensitizers on nanoparticles^11^ or cross-linking,^12^ enabled the recycling of these photocatalysts, promoting a more environmentally friendly process. This goal was also achieved by using nature-derived photocatalysts, such as sodium pyruvate,^13,14^ chlorophyll a,^15^ and carbon dots.^16,17^ Additionally, the use of porphyrinic metal–organic framework nanosheets for 3D printing enhanced the mechanical properties and antibacterial activity of resulting materials.^18^ Moreover, integrating photosensitizers into the thermoresponsive hydrogel facilitated the regulation of polymerization by both light and temperature.^19^ The combination of a photocatalytic system with DNA nanotechnology also shows promise.^20−22^ For example, a photocatalyst and a quencher were incorporated within self-assembled DNA nanostructures.^20^ An introduction of chemical stimuli induced a conformational change in the DNA structure, disrupting the proximity between the photocatalyst and quencher and activating the photocatalyst for RDRP.

As a step toward expanding the horizons of photocatalytic platforms for photopolymerizations, we sought novel photocatalysts that have been underexplored in the field of RDRP. Among various dyes and photocatalysts, nucleic acid-binding dyes (**NuABDs**) caught our attention. **NuABDs** are interesting fluorescent dyes which interact with nucleic acids through mechanisms such as intercalation and groove-binding.^23,24^ Importantly, upon binding to nucleic acids, **NuABDs** often exhibit a significantly enhanced fluorescence. This fluorogenicity is attributed to the prolonged lifetimes of **NuABD** in the excited state (NuABD*) caused by restricted photoisomerization of the monomethine bridge (for nonsymmetric cyanine dyes)^24,25^ or the delayed proton transfer to the solvents (for ethidium bromide).^26^ Due to their unique properties, **NuABDs** have been widely utilized in nucleic acid engineering as a light-up probe for diagnostic and analytical applications. Nonetheless, the photocatalytic activity of **NuABDs** as a photocatalyst has not yet been systemically explored. This study demonstrates the first example of a photocatalytic platform for the photoATRP process that utilizes **NuABDs** as a versatile photocatalyst.

**Scheme 1:**
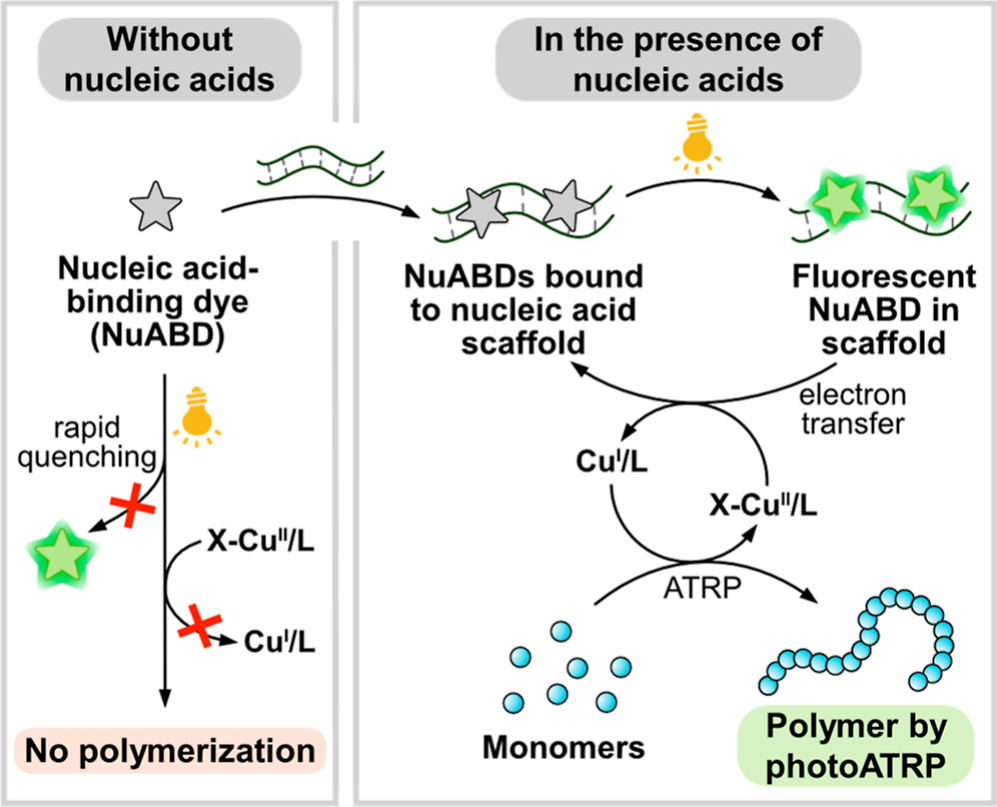
PhotoATRP Mediated by **NuABDs** as the Photocatalyst in the Presence of Nucleic Acids

Distinguished from previous photoATRP techniques, our **NuABD**-based photocatalytic system was active exclusively in the presence of nucleic acid scaffolds (Scheme 1). Following the binding to the scaffolds, the increased lifetime and higher quantum yields of NuABD* under light irradiation enabled efficient electron transfer from NuABD* to the X–Cu^II^/L ATRP catalyst (X = Br or Cl, L = ligand).^27,28^ Subsequently, the reduced catalyst (Cu^I^/L) generated propagating radicals through the cleavage of the carbon–halogen bond in the ATRP initiator. After the addition of a few monomer units, the propagating radical is deactivated by the X–Cu^II^/L catalyst and goes back to a dormant state, which later can be reactivated by Cu^I^/L. Conversely, in the absence of nucleic acids, the rapid fluorescence quenching of **NuABDs** hindered the efficient reduction of the X–Cu^II^/L catalyst, inhibiting polymerizations. We envisioned that by leveraging DNA-related techniques such as self-assembly and enzymatic DNA amplification, the **NuABD**-based photopolymerization system could offer an intriguing route for developing multiresponsive RDRP platforms or facilitating nanofabrication and efficient polymerization under biofriendly environment.^29−31^

## Results and Discussion

### Investigating the Photocatalytic Activity of NuABDs

We started with the investigation of interactions between **NuABDs** and readily available nucleic acids extracted from biomass (salmon DNA and yeast RNA). We selected popular nontoxic intercalating dyes (GelGreen and GelRed, the homodimer of acridine orange and ethidium bromide, respectively) and a nonsymmetric cyanine dye (SYBR Gold). The change in the absorption spectra of **NuABDs** upon the addition of DNA or RNA (Figure S1) indicated their successful binding to both nucleic acids. Consequently, significant fluorescence of **NuABDs** was observed only in the presence of nucleic acids due to the fluorogenicity of **NuABDs** upon binding to nucleic acids (Figure S2). Notably, the increase in fluorescence intensity was greater with salmon DNA compared to yeast RNA. This is due to the higher affinities of **NuABDs** for salmon DNA which exhibit higher molecular weights and a duplex structure facilitating the dyes’ intercalation (Figure S3).^23^

**Figure 1:**
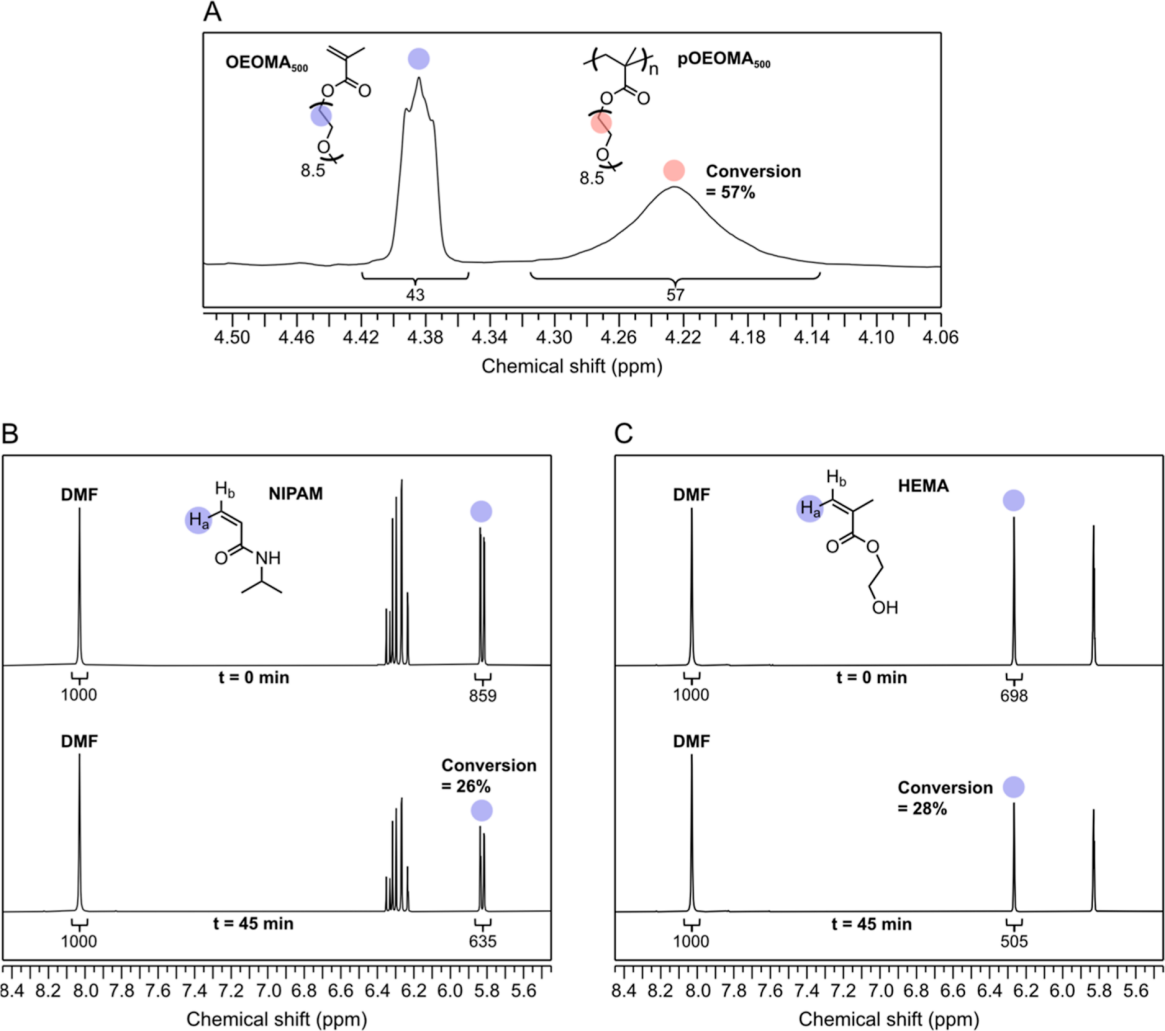
Representative ^1^H NMR spectra for the determination of monomer conversion. (A) Conversion of OEOMA_500_ was determined by comparing areas of the monomer peak (blue circle, ca. 4.38 ppm) and polymer (red circle, ca. 4.22 ppm). (B) The conversion of NIPAM was determined by using DMF as the internal standard and monitoring the decrease in the area corresponding to a proton in NIPAM (blue circle, ca. 5.8 ppm). (C) The conversion of HEMA was determined by using DMF as the internal standard and monitoring the decrease in the area corresponding to a proton in HEMA (blue circle, ca. 6.25 ppm).

Next, we proceeded to assess the potential of **NuABDs** as photocatalysts for mediating ATRP (Table 1). Polymerizations were conducted in phosphate-buffered saline (PBS) without deoxygenation under the irradiation of light (λ = 520 nm, 3.7 mW cm^–2^), using a custom photoreactor (Figure S4). We wanted to avoid the use of UV light (λ < 350 nm), which could cause side reactions by directly generating radicals through the cleavage of alkyl halide bond or the decomposition of ATRP ligand. 2-Hydroxyethyl 2-bromoisobutyrate (HEBiB) and tris(2-pyridylmethyl)amine (TPMA) were utilized as the initiator and the ligand, respectively (see Figure S4F for chemical structures). As a model monomer, a PEG-like monomer (i.e., OEOMA_500_ = oligo(ethylene oxide) methyl ether methacrylate, average *M*_n_ of 500) was employed. Monomer conversions were determined by ^1^H NMR spectroscopy (Figure 1). As indicated in Table 1, no polymerization occurred in the absence of nucleic acid (entry 1), **NuABD** (entry 2), or the Cu^II^/TPMA complex (entry 3). In contrast, successful polymerizations were observed when salmon DNA was used along with GelGreen and the Cu^II^/TPMA complex (entries 4–6). It should be noted that the use of excess TPMA ligands was crucial, and no polymerization was observed without the addition of excess TPMA (Table S2). This observation aligns well with previously reported photoATRP systems based on eosin y^27^ or methylene blue^28^ where N-based TPMA played an important role as both ligand and electron donor to regenerate the photocatalyst in the oxidative quenching cycle (entries 1–3 in Table S2). We also would like to highlight that when excess electron donor (i.e., TPMA) was replaced with DNA, negligible conversion was observed, indicating that electron transfer from DNA to Cu^II^/TPMA or oxidized **NuABD** is not favored (entries 3 and 4 in Table S2). Additionally, the negligible conversion in the absence of Cu^II^/TPMA complex (entry 3 in Table 1) highlights that GelGreen and GelRed are mild photocatalysts that predominantly donate electrons to copper catalysts without directly generating radicals.

**Table 1:** PhotoATRP Mediated by **NuABDs** in the Presence of Nucleic Acids^a^

entry	nucleic acid (concentration)	**NuABD** (concentration)^b^	conv.^c^ (%)	*M*_n,NMR_^d^	*M*_n,GPC_^e^	*M*_n,Abs_^f^	*D̵*^e^
1		GelRed or GelGreen (10×)	0				
2	salmon DNA (0.1 mg/mL)		0				
3^g^	salmon DNA (0.1 mg/mL)	GelRed or GelGreen (10×)	0				
4	salmon DNA (0.02 mg/mL)	GelGreen (10×)	13	19,500	24,800	26,200	1.16
5	salmon DNA (0.1 mg/mL)	GelGreen (10×)	57	85,500	64,200	82,900	1.09
6	salmon DNA (0.5 mg/mL)	GelGreen (10×)	77	115,500	79,700	107,800	1.12
7	salmon DNA (0.02 mg/mL)	GelRed (10×)	8	12,000	17,700	17,400	1.17
8	salmon DNA (0.1 mg/mL)	GelRed (10×)	49	73,500	86,400	118,800	1.10
9	salmon DNA (0.5 mg/mL)	GelRed (10×)	55	82,500	92,900	129,800	1.09
10	salmon DNA (0.1 mg/mL)	GelGreen (3×)	2				
11	salmon DNA (0.1 mg/mL)	GelGreen (30×)	85	127,500	84,500	115,700	1.17
12		SYBR Gold (10× or 20×)	0				
13	salmon DNA (0.1 mg/mL)	SYBR Gold (10×)	6	9000	16,200	15,600	1.12
14	salmon DNA (0.1 mg/mL)	SYBR Gold (20×)	50	75,000	58,900	74,800	1.11
15	yeast RNA (0.1 mg/mL)	GelGreen (10×)	0				
16	yeast RNA (2.5 mg/mL)	GelGreen (10×)	18	27,000	28,200	30,600	1.12
17	yeast RNA (2.5 mg/mL)	GelGreen (30×)	36	54,000	43,200	51,400	1.13
18	dNTP (0.3 mM)	GelGreen (10×)	0				
19	ssDNA, 18-mer (0.1 mg/mL)	GelGreen (10×)	0				
20	ssDNA, 92-mer (0.1 mg/mL)	GelGreen (10×)	50	75,000	62,300	79,900	1.09
21	bulged stem-loop DNA, 35-mer (0.1 mg/mL)	GelGreen (10×)	67	100,500	76,900	103,200	1.12

a Reaction conditions: [OEOMA_500_] = 360 mM, [OEOMA_500_]/[HEBiB]/[CuBr_2_]/[TPMA] = 300/1/0.75/2.25. The reactions were performed in PBS under light (λ = 520 nm, 3.7 mW cm^–2^) for 45 min. Oligonucleotide sequences for entries 18–21 are shown in Table S1.

b The molarity of GelGreen, GelRed, and SYBR Gold (commercially sold as 10,000× concentrate) are estimated to be 40.7, 31.3, and 20.1 mM, respectively. See Table S9.

c Monomer conversions determined by ^1^H NMR spectroscopy.

d Theoretical molecular weight calculated from conversion assuming quantitative initiation.

e Molecular weight and dispersity determined by using GPC (DMF as eluent) calibrated with PMMA standards. GPC traces of entries 4–17 are shown in Figure S5. GPC traces and schematic illustrations of oligonucleotides used for entries 18–21 are given in Figure S8.

f Molecular weight determined by Mark–Houwink calibration following the previously reported procedure, to compensate hydrodynamic volume difference.^27,32^

g The reaction performed without CuBr_2_ and TPMA ligand.

We noticed increased monomer conversions with higher amounts of DNA (entries 4–6 in Table 1), resulting from enhanced binding of **NuABDs**. This trend was also observed when GelGreen was replaced by GelRed (entries 7–9 in Table 1). At constant DNA concentration, higher dye loading resulted in increased monomer conversion (entries 5, 10, and 11 in Table 1). In addition to GelGreen and GelRed, the nonsymmetric cyanine dye (SYBR Gold) was also tested (entries 12–14 in Table 1). The good agreement between the theoretical molecular weight (*M*_n,NMR_) and the number-averaged absolute molecular weight determined by Mark–Houwink calibration (*M*_n,abs_)^27,32^ confirmed that SYBR Gold is also a promising photocatalyst (entry 14 in Table 1). Notably, the fraction of GelRed and SYBR Gold bound to salmon DNA was calculated, based on the McGhee-von Hippel model of ligand-substrate binding (see the Supporting Information discussion).^33^ As shown in Table S10, the higher binding of **NuABD** to salmon DNA was correlated with efficient polymerization, implying that the bound dye predominantly facilitates polymerization due to its enhanced photophysical properties.

Next, we attempted to employ the **NuABD**-based photocatalytic system for RAFT polymerization (entries 1–4 in Table S3). However, no polymerization was observed. This is due to the much more negative reduction potentials of CTA (ca. −1.2 V vs SCE)^34^ than those of Cu^II^/TPMA complex (ca. −0.3 V vs SCE). As previously observed in other photocatalytic systems, this leads to much less efficient electron transfer from the excited photocatalyst to the RAFT CTA.^27,28,34,35^

We conducted further polymerizations using yeast RNA (Figure S3), a shorter and single stranded scaffold compared to salmon DNA (entries 12 and 13 in Table 1). Due to the less efficient binding of **NuABDs** to yeast RNA, up to 36% conversion was achieved after polymerization with a relatively large amount of RNA (2.5 mg/mL) in the presence of 10× or 30× GelGreen (entries 16 and 17 in Table 1). This inspired us to investigate the effect of nucleic acid length on the polymerization efficiency using oligonucleotides with different molecular weights and structures. Interestingly, no polymerization was observed when the monomeric unit of DNA (deoxyribonucleoside triphosphate, dNTP, entry 18 in Table 1) or single-stranded 18-mer DNA (ssDNA, entry 19 in Table 1) was used because of less-efficient binding of **NuABDs** to these scaffolds. In contrast, successful polymerization was achieved (conversion of 50%) when 92-mer ssDNA was employed as the scaffold (entry 20 in Table 1). Additionally, we performed a polymerization using 35-mer DNA, which self-assembles into a bulged stem-loop structure, as shown in Figure S8. Interestingly, despite its relatively shorter length, an increased conversion of 67% was observed (entry 21 in Table 1). This could be attributed to the intercalation as the dominant binding mode of GelGreen, which preferentially occurs at the double-stranded region in the structure.^23,36^ These results imply that the length and secondary structure of nucleic acid scaffolds would be important factors for the well-controlled polymerization process. Due to the low amount of DMSO used (<1% *v*/*v*, originating from TPMA and **NuABD** stocks), the change in the secondary structure of nucleic acids is not likely under standard polymerization conditions. However, the use of hydrophobic monomers (e.g., styrene) or intercalating ligands may lead to structural changes in the nucleic acids.^37,38^ Additionally, due to the use of green light, the effect of light irradiation on the secondary structure of nucleic acids is mitigated.^39^

In addition to OEOMA_500_, we investigated additional monomers: 2-hydroxyethyl methacrylate (HEMA) and *N*-isopropylacrylamide (NIPAM). Of note, NIPAM was polymerized using tris[2-(dimethylamino)ethyl]amine (Me_6_TREN) as the ligand, which is suitable for acrylamide derivatives.^40^ Polymerization was performed for 45 min in PBS under green light using 0.1 mg/mL salmon DNA and Gelgreen (10×). Monomer conversions were determined by ^1^H NMR using dimethylformamide (DMF) as the internal standard (Figure 1B,C). Moderate conversions, narrow molecular-weight distributions, and symmetric GPC traces were observed (Figure S9), indicating successful polymerization of NIPAM and HEMA, in addition to the OEOMA_500_.

### Proposed Mechanism of the NuABD-Mediated Photocatalysis

After establishing that the formation of dye-nucleic acid complexes is fundamental for promoting polymerization, we investigated the role and the thermodynamics of the polymerization components in order to propose a comprehensive mechanism for the **NuABD**-mediated photo ATRP.

**Figure 2:**
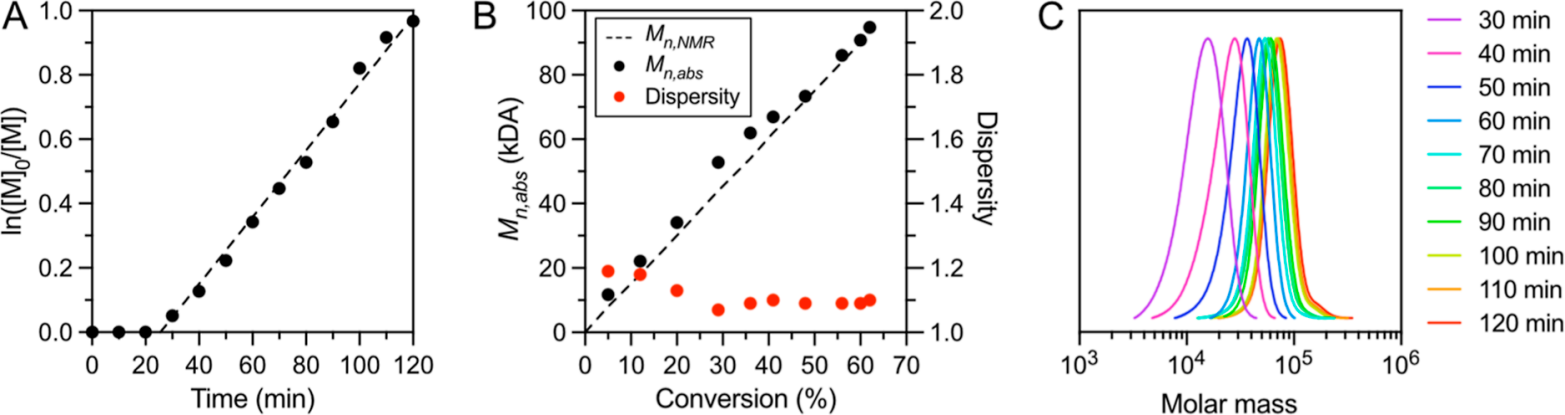
Analysis of polymerization kinetics using salmon DNA (0.1 mg/mL) and GelGreen (10×). (A) First-order kinetic plot of ATRP of OEOMA_500_. (B) Evolution of molecular weight (*M*_n,abs_) and dispersity with monomer conversion. (C) GPC traces at each time point. Polymerization results and reaction conditions are presented in Table S4.

The excited state **NuABDs** can react with the copper catalyst through either a reductive or an oxidative quenching pathway, discernible by evaluating the relative thermodynamics of the two pathways (Figure 2A,B). We first evaluated the redox potentials of all species involved in the photoredox cycle from cyclic voltammetry analysis and from the literature data (see Table S8). Under green light irradiation, the photocatalyst GelGreen (GG) in the excited state (GG*) can both accept [*E*°(GG*/GG^•–^) = +1.20 V vs SCE] or donate an electron [*E*°(GG^•+^/GG) = −1.57 V vs SCE]. In the reductive quenching cycle, GG* is reduced by accepting an electron from the excess of the ATRP ligand, which bears a tertiary nitrogen atom and acts as a sacrificial electron donor. This results in the formation of the GG radical anion (GG^•–^) and an amine radical cation (L^•+^). The formed GG^•–^ [*E*°(GG/GG^•–^) = −1.13 V vs SCE] then donates an electron to Cl–Cu^II^/L, generating the Cu^I^/L activator and regenerating GG in the ground state, completing the photocatalytic cycle. Conversely, in the oxidative quenching cycle, GG* is oxidized by donating an electron to Cl–Cu^II^/L, leading to the formation of GG^•+^ and Cu^I^/L. Finally, the photocatalytic cycle is closed by reducing the oxidized GG^•+^ [*E*°(GG^•+^/GG) = +0.76 V vs SCE] with an alkylamine.

The free energy of a photoinduced electron transfer can be determined from the following equation
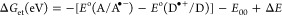
1where *E*°(A/A^•–^) is the standard potential of an electron acceptor (A), *E*°(D^•+^/D) is the standard potential of a sacrificial electron donor (D), *E*_00_ is the energy of the singlet or triplet excited state of a photocatalyst, and Δ*E* < 0.1 eV is a Coulombic contribution that is often negligible in photophysical estimations. For GG, the excitation energy is 2.33 eV. Thus, for the oxidative quenching
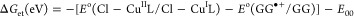
2where *E*°(Cl–Cu^II^L/Cl–Cu^I^L) = −0.32 V vs SCE.^41^ Therefore, the Δ*G*_et_ for oxidative quenching is −1.25 eV (−28.8 kcal mol^–1^). In contrast, for the reductive quenching
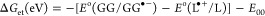
3

**Scheme 2:**
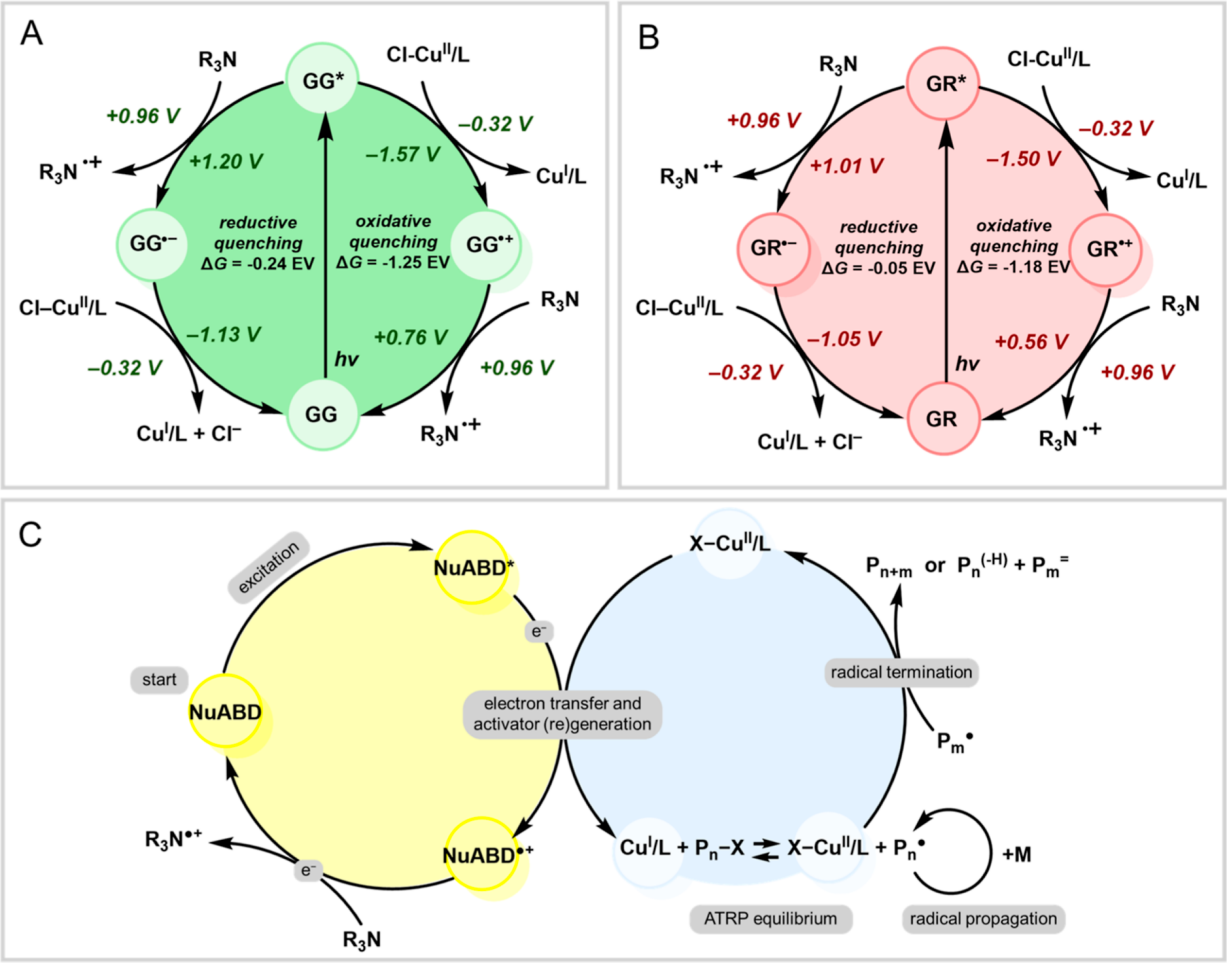
(A,B) Reductive Quenching vs Oxidative Quenching Cycle for (A) GelGreen; and (B) GelRed; And (C) Proposed Mechanism of photoATRP Using **NuABD** as the Photocatalyst Note that Cl–Cu^II^L, instead of Br–Cu^II^L, is the dominant Cu(II) complex due to the presence of excess chloride anions in PBS solution.

Assuming that the redox potential of the TPMA ligand is close to that of Et_3_N [*E*°(Et_3_N^•+^/Et_3_N) = −0.96 V vs SCE], Δ*G* can be estimated as −0.24 eV (−5.5 kcal mol^–1^). Despite some approximations (Table S8 and Supporting Information discussion), these thermodynamic calculations clearly indicate that the oxidative quenching of GG* is the most favorable pathway to reduce Cu^II^ to Cu^I^, sustaining the polymerization process (Scheme 2A). A similar rationale can be extended to GelRed (GR), which yields comparable results that were obtained with the GR photocatalysts, for which the oxidative quenching pathway is also strongly favored (Δ*G*_et_ = −1.18 eV or −27.2 kcal mol^–1^, Scheme 2B).

Fluorescence quenching experiments (Figure S16) provided further experimental support for the oxidative quenching pathway. The quenching experiments revealed a significant reduction in the fluorescence intensity of GelGreen when the CuBr_2_/TPMA complex was introduced (Figure S16A), whereas only a negligible change in fluorescence occurred with the addition of the TPMA ligand, without CuBr_2_ (Figure S16B). Based on the evidence obtained from cyclic voltammetry investigations, fluorescence quenching experiments, and the polymerization with various amounts of amine (Table S2), it is concluded that this photopolymerization system proceeds via an oxidative quenching cycle, similar to previously reported ATRP systems (Scheme 2C).^27,28,42^

In contrast, this oxidative quenching process was unable to sustain a RAFT polymerization. In the RAFT process (Table S3), the reduction of a RAFT chain end is required, having a much more negative reduction potential of ca. −1.2 V vs SCE,^43^ in contrast to copper complexes (ca. −0.3 V vs SCE). In the RAFT scenario, the oxidative quenching pathway (eq 2) is considerably less favored, with a value of Δ*G*_et_ = −0.37 eV (−8.5 kcal mol^–1^). This low thermodynamic driving force is likely the cause of the slow photoinduced electron (or energy) transfer between excited photocatalysts and the RAFT agents, leading to the absence of RAFT polymerization.

### Evidence of Controlled Polymerization

Next, we investigated the kinetics of the **NuABD**-mediated photoATRP process to confirm controlled radical polymerization behavior (Figure 2). Initially, a short induction period was observed. This period is attributed to the deoxygenation process during which triplet oxygen is scavenged by excited photocatalysts or the Cu^I^/TPMA complex. The oxidized complex could then be subsequently regenerated through electron transfer from the photocatalysts.^44^ After the induction period, semilogarithmic monomer consumption (Figures 2A and S10) and evolution of molecular weight with conversion (*M*_n,Abs_ in Figure 2B) both exhibited linearity, maintaining a low dispersity. A good agreement between *M*_n,NMR_ and *M*_n,abs_ (Figure 2B and Table S4), and symmetrical monomodal GPC traces (Figure 2C) were recorded. Moreover, we found good control of polymer molecular weights up to over 100,000, and good temporal control upon switching the light on and off (Table S5 and Figure S11), confirming the role of **NuABDs** as a promising photocatalyst for well-controlled ATRP. It is noteworthy that a slight increase in conversion (1–2%) was observed during dark periods in the temporal control experiments (Figure S11B). This is attributed to the presence of residual ATRP activator (i.e., Cu^I^/TPMA) which is often observed in photoATRP systems or even in photo click reactions mediated by copper.^45^

### Broadening the Scope of Photocatalysts

**Figure 3:**
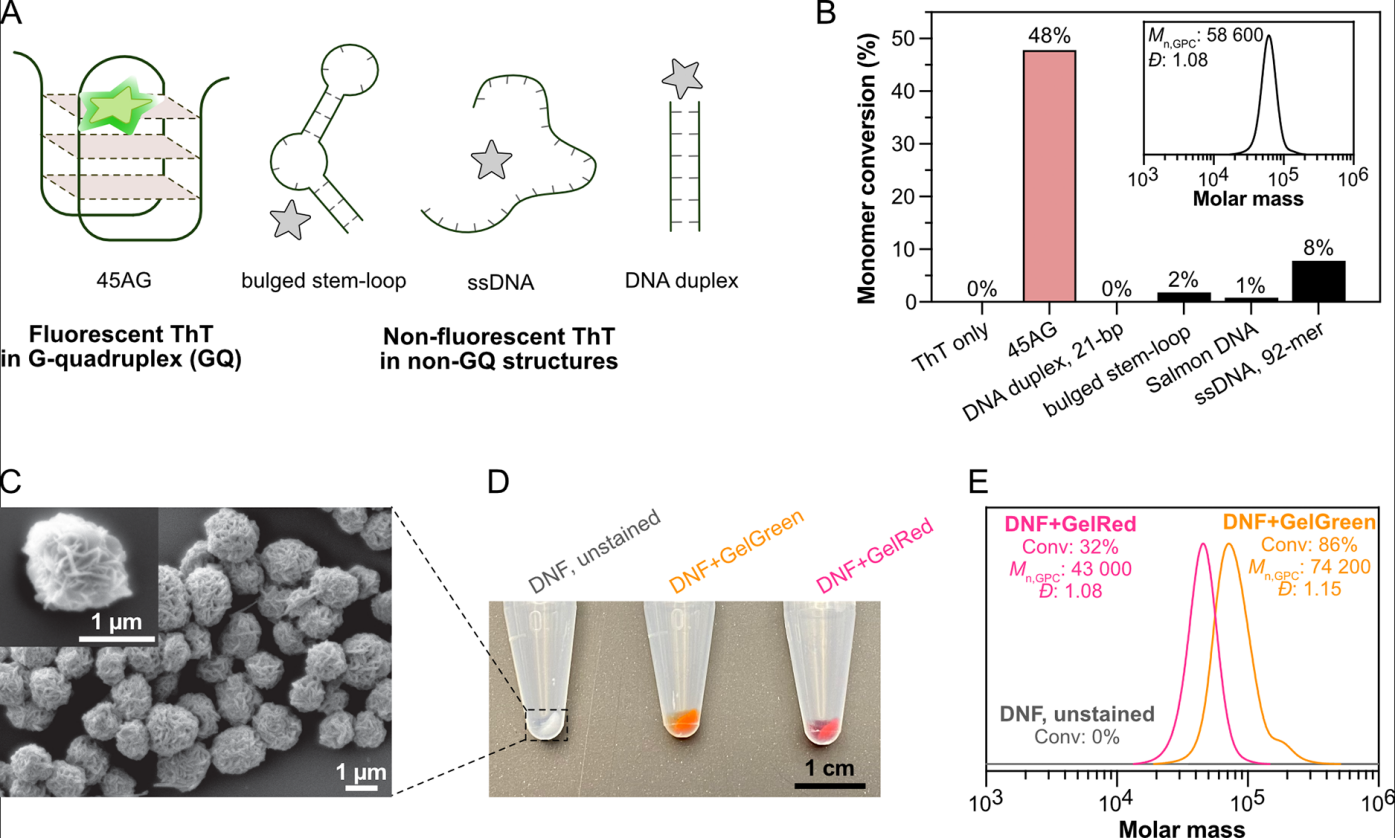
PhotoATRP using DNA-based nanostructures. (A) Light emission from ThT in the presence of the G-quadruplex. (B) Monomer conversion after photoATRP using ThT in the presence of different DNA scaffolds. Inset: GPC trace after photoATRP using 45AG and ThT under blue light (λ = 450 nm, 5.8 mW cm^–2^) for 45 min in PBS. The polymerization results are summarized in Table S6. (C) SEM image of the DNFs. Inset: higher magnification (35,000×) image of a DNF. (D) Digital camera image of the DNF pellet before and after staining. (E) GPC traces after photoATRP using DNFs. The polymerization results are summarized in Table S7.

In addition to the already discussed **NuABDs**, which can bind to a broad range of nucleic acids, there are unique **NuABDs** that light up after binding to specific nucleic acids, forming appropriate secondary structures. Due to their specificity, such **NuABDs** have gathered significant attention as fluorescent probes for diagnostic and analytical applications (e.g., real-time quantitative polymerase chain reaction) as well as DNA origami-based nanopatterning.^22,46^ To demonstrate the versatility of our **NuABD**-based photocatalytic system, we utilized thioflavin T (ThT) as a photocatalyst for ATRP under blue light (λ = 450 nm, 5.8 mW cm^–2^). ThT is known to selectively bind to the specific DNA sequences forming G-quadruplex (GQ) structures and exhibit an enhanced quantum yield (Figures 3A and S12).^47,48^ Successful polymerization was observed only when ThT was utilized along with a GQ-forming DNA scaffold (i.e., 45AG, from human telomeric DNA), as shown in Figure 3B. In contrast, negligible conversions were obtained when 45AG was replaced with alternative DNA sequences and structures. This implies the potential of the **NuABD**-based photoATRP platform to be selectively activated in the presence of desired **NuABDs** and appropriate nucleic acids.^49^

Until recently, a variety of multiscale nucleic acid-based structures have been reported, holding promise for diverse applications.^50−53^ To broaden the scope of nucleic acid scaffolds, we sought to explore three-dimensional DNA-based materials. We chose DNA nanoflowers (DNFs),^54−61^ a well-studied theragnostic agent, because DNFs can be labeled with and can increase the local concentration of functional molecules. Thus, we aimed to investigate whether NuABDs could mediate polymerization when encapsulated locally within DNFs rather than when being homogeneously dispersed. Micron-sized DNFs were enzymatically synthesized, as previously reported (Figures 3C and S14A).^54,56^ The resulting DNFs were stained with **NuABDs** (GelGreen or GelRed), and residual **NuABDs** were removed by three consecutive centrifugations. Successful staining of DNFs was confirmed by digital camera images of DNFs in a pellet (Figure 3D) and suspension (Figure S14B), as well as fluorescence images (Figure S14C–E).

The stained DNFs underwent brief sonication to prepare well-dispersed DNFs, which may have aggregated after centrifugations. The DNFs were mixed with the prepolymerization mixture, followed by 15 min of argon purging to remove excess oxygen in the empty space (Figure S4D,E). After 45 min of polymerization with continuous stirring in a 1.85 mL vial, DNFs were removed by centrifugation, and the supernatants were subjected to GPC analysis. As shown in Figure 3E, significant conversion was achieved only when stained DNFs were utilized as the photocatalyst, and the resulting polymer exhibited low dispersity. This highlights that, in addition to linear or planar DNA nanostructures, three-dimensional DNA-based materials with locally concentrated **NuABDs** could also function as an efficient photocatalytic platform. We also performed polymerization using a supernatant of sonicated DNF after centrifugation (Figure S15) to examine the leaking of DNA from DNF after sonication. Considering the transparent color of supernatants (Figure S15A) and negligible conversion after polymerizations using the supernatants (Figure S15B), it can be inferred that DNA leaching is negligible and that the majority of DNA and **NuABD** remain within the DNF structures. It is noteworthy that while the dye thoroughly stained the particles, the well-controlled ATRP was facilitated by the water-soluble Cu catalyst, which was not scavenged by the bulky DNA structures.^32,45^

## Conclusions

In conclusion, we have demonstrated that **NuABDs** together with appropriate nucleic acid scaffolds are promising photosensitizers for photoATRP. The developed **NuABD**-based photocatalytic system reveals exclusive activity in the presence of nucleic acids, providing a versatile platform for controlled polymerization processes. Additionally, enhanced conversion was achieved in the presence of long double-stranded scaffolds compared to that of short single-stranded scaffolds. Controlled polymerization behavior and excellent temporal control were observed without the need for deoxygenation, which is beneficial for polymerization in the presence of biomolecules.^62,63^ Under 450 nm irradiation, ThT gave significant polymerization exclusively in the presence of G-quadruplexes. The versatility of this system was further demonstrated through successful polymerization with multiscale nucleic acid scaffolds, highlighting its potential for applications in materials science and biotechnology. By leveraging programmable self-assembly (e.g., toehold mediated strand displacement) and nucleic acid amplification techniques (e.g., polymerase chain reaction and rolling circle amplification),^64−68^ we anticipate that the **NuABD**-based photocatalytic ATRP platform would open new opportunities such as stimuli-responsive polymerization,^19,29^ nanofabrication,^22,46^ and nucleic acid–polymer biohybrids.^32,64,66,67^

## Supporting Information Available

Absorbance spectra, measurements of fluorescence intensity, GPC traces, polymerization setup, ^1^H NMR spectra, sequences of oligonucleotides, polymerization results, DLS result, and fluorescence microscopy (PDF)
