# Supplementary material for: Nucleic Acid-Binding Dyes as Versatile Photocatalysts for Atom-Transfer Radical Polymerization
Source: J Am Chem Soc. 2024 May 1;146(19):13598–606. doi: 10.1021/jacs.4c03513 (PMC11100002; doi:10.1021/jacs.4c03513)
Supplement: Supplementary file 1 — ja4c03513_si_001.pdf [file ja4c03513_si_001.pdf]

Supporting Information

Nucleic Acid Binding Dyes as Versatile Photocatalysts for Atom Transfer Radical Polymerization

Jaepil Jeong,[1,2] Xiaolei Hu,[1] Rongguan Yin,[1] Marco Fantin,[3]* Subha R. Das,[1,2]* and Krzysztof Matyjaszewski,[1]*

[1]Department of Chemistry, Carnegie Mellon University, Pittsburgh, Pennsylvania 15213, United States.

[2]Center for Nucleic Acids Science & Technology, Carnegie Mellon University, Pittsburgh, Pennsylvania 15213, United States

[3]Department of Chemical Sciences, University of Padova, Via Marzolo 1, 35131 Padova, Italy

***Correspondence:** M.F. (marco.fantin@unipd.it)
S.R.D. (srdas@andrew.cmu.edu)
K.M. (km3b@andrew.cmu.edu)

Table of Contents

Experimental details 3
Materials 3
Instruments 3
Cyclic voltammetry (CV) 3
Preparation of stock solutions 4
Characterization of nucleic acid-dye complexes using microplate reader 4
General polymerization procedure 5
PET-RAFT polymerization 5
Photo ATRP using oligonucleotide as the co-catalyst 5
Analysis of polymerization kinetics 6
Temporal control 6
Photo ATRP using G-quadruplex forming DNA and Thioflavin T 6
Photo ATRP using DNA nanoflower as the co-catalyst 7
Estimation of absolute concentration 7
Supplementary Figures 8
Supplementary Tables 24
Supplementary Discussions 34
Cyclic voltammetry analysis of NuABDs 34
Effect of DNA binding on the photophysical properties of the NuABDs 35
Additional mechanistic discussions 35
Quantification of GelRed bound to DNA 35
References 36

Experimental details

Materials

Oligo(ethylene glycol) methyl ether methacrylate (average M_n 500, $OEOMA_{500}$), 2-hydroxyethyl methacrylate (HEMA), *N*-isopropylacrylamide (NIPAM) and 2-hydroxyethyl 2-bromoisobutyrate (HEBiB) were purchased from Sigma-Aldrich. GelGreen and GelRed (10000x concentrate in DMSO) were purchased from Biotium. SYBR Gold (10000x concentrate in DMSO) was purchased from Invitrogen. 10x phosphate-buffered saline (PBS) were purchased from Fisher Scientific. Tris(2-pyridylmethyl)amine (TPMA) was purchased from TCI America. G-quadruplex forming oligonucleotide (45AG) was purchased from Sigma-Aldrich. All other oligonucleotides were purchased from Integrated DNA Technologies (IDT). T4 DNA ligase and 10x ligase buffer were purchased from Promega. phi29 DNA polymerase and 10x phi29 DNA polymerase buffer were purchased from Nxgen. Deoxynucleotide (dNTP) solution mix was purchased from New England Biolabs. 250 μL glass inserts were purchased from Restek. LED strips (12VDC) for the fabrication of photoreactor were purchased from aspectLED and the strips were mounted inside a glass jar (diameter = 9 cm, height = 7 cm). Me_6TREN (*N*,*N*,*N*′,*N*″,*N*″- tris[2-(dimethylamino)ethyl]amine) was received from Koei Chemical Co., Ltd. All other chemicals, including DNA sodium salt from salmon testes (D1626) and torula yeast RNA (R6625), were purchased from Sigma-Aldrich unless stated otherwise.

Instruments

^1H nuclear magnetic resonance (NMR) spectra were recorded using Bruker Avance III 500 MHz spectrometer (Bruker). The absorbance spectra and fluorescence intensities of dyes were measured using Infinite® M1000 microplate reader (Tecan). FEI Quanta 600 FEG Scanning Electron Microscope (SEM) was adopted for the characterization of DNA nanoflowers. For the characterization of polymerization products, Waters 515 HPLC pump equipped with Waters 2414 refractive index detector, and PSS GRAM columns were used in DMF as eluent at a flow rate of 1 ml/min at 50°C. The GPC was calibrated with poly(methyl methacrylate) (PMMA) standards to determine apparent molecular weights ($M_{n,GPC}$) and dispersity (M_w/M_n). Characterization of biomass DNA (from Salmon) and RNA (from torula yeast) was performed by Agilent 1260 Infinity II module with UV detector, multi-angle light scattering detector (DAWN, Wyatt), viscometer (ViscoStar, Wyatt), refractive index detector (Optilab, Wyatt), and Agilent Suprema column using 0.1 M Dulbecco's phosphate buffer saline (DPBS) as a running buffer at a flow rate of 0.5 ml/min at 30°C. Zetasizer Nano ZS (Malvern) was used for dynamic light scattering (DLS) analysis of DNA nanoflowers. Zeiss 880 Laser Scanning Confocal Inverted Microscope was used for the fluorescence imaging of DNA nanoflowers.

Cyclic voltammetry (CV)

CVs were performed by an Autolab PGSTAT30 potentiostat (EcoChimie, Utrecht, The Netherlands) interfaced to a PC running an Autolab GPES 4.9 software. A three-electrode cell with a glassy carbon working

electrode (3 mm diameter disc, Metrohm) and a Pt counter electrode was used. The reference electrode was a saturated calomel reference electrode (SCE, Schott Gerade). The surface of the working electrode was polished with 0.25 μm diamond paste followed by sonication in ethanol before each CV.

Preparation of stock solutions

Stock solutions for the reactions are prepared as follows using nuclease free water or Milli-Q water.

0.6 M $OEOMA_{500}$ stock	:	3000 mg of $OEOMA_{500}$ in 10 mL of water
0.6 M NIPAM stock	:	67.9 mg of NIPAM in 1 mL of water
0.6 M HEMA stock	:	78.1 mg of HEMA in 1 mL of water
100 mM HEBiB stock	:	10.6 mg of HEBiB in 0.5 mL of water
56.2 mM $CuBr_2$ stock	:	25.1 mg of $CuBr_2$ in 2 mL of 50% *v*/*v* DMSO in water
336.3 mM TPMA stock:	:	24.4 mg of TPMA in 0.25 mL of DMSO
100 mM CPADB stock	:	14.0 mg of CPADB in 0.5 mL of DMSO
100 mM TEOA stock	:	14.9 mg of TEOA in 1 mL of water
1.5 mM eosin y stock	:	4.86 mg of eosin y in 5 mL of 10% *v*/*v* DMSO in water
1000x NuABD stock	:	50 μL of the dye (10000x concentrate in DMSO) in 450 μL of water
2 mg/mL salmon DNA stock	:	2 mg of DNA sodium salt from salmon testes in 1 mL of water
8 mg/mL Torula yeast RNA stock	:	8 mg of Torula yeast RNA in 1 mL of water
200 μM oligonucleotides	:	DNA pellets were dissolved in (final conc. of 200 μM)
4.5 mM Thioflavin T (ThT) stock	:	7.17 mg of ThT in 5 mL of 10% *v*/*v* DMSO in water

Prior to the preparation of $OEOMA_{500}$ or HEMA stock, crude $OEOMA_{500}$ or HEMA were passed through basic alumina to remove inhibitors. NIPAM was recrystallized three times from n-hexane prior to use for the removal of inhibitors. To make yeast RNA stock, the mixture of RNA and water underwent sonication for 20 min to prepare homogeneous suspension. 21-bp DNA duplex was prepared by heating two complementary DNA strands (90 μM each, see Entry 6 in Table S1 for sequences) in 1x PBS at 90°C for 8 min followed by cooling in ice bath for 10 min.

Characterization of nucleic acid-dye complexes using microplate reader

20 μL of a 10x phosphate-buffered saline (PBS) solution, 2 μL of a 1000x dye stock solution, and either salmon DNA or yeast RNA stock (at the final concentration ranging from 0 to 0.5 mg/mL) were combined. The total volume was adjusted to 200 μL by adding water. Subsequently, the resulting mixtures, each containing different nucleic acid concentrations, were transferred into a 96-well plate. Absorbance spectra and fluorescence intensities of each well were recorded using a microplate reader. For the measurement of fluorescence intensity, the following

excitation and emission wavelengths were employed for the respective dyes: GelGreen (λ_{ex} = 510 nm, λ_{em} = 528 nm), GelRed (λ_{ex} = 526 nm, λ_{em} = 600 nm), and SYBR Gold (λ_{ex} = 495 nm, λ_{em} = 538 nm). Quenching experiments were performed in 96-well plate by stepwise addition of $CuBr_2$/TPMA stock (1:1, 48.3 mM each) or TPMA stock (96.6 mM) to the salmon DNA (0.1 mg/mL) with GelGreen (10x) in PBS, at r.t.

General polymerization procedure

For the polymerization under the standard condition (0.1 mg/mL of salmon DNA with 10x GelGreen, 10x GelRed, or 20x SYBR Gold) at the target DP of 300, 150 μL of $OEOMA_{500}$ stock, 3 μL of HEBiB stock, 4 μL of $CuBr_2$ stock, 2 μL of TPMA stock, 25 μL of 10X PBS, 12.5 μL of salmon DNA stock, 2.5 μL of GelGreen or GelRed stock were mixed and the final volume was brought to 250 μL by adding water. The reaction mixture was thoroughly mixed and transferred into a 250 μL glass insert followed by irradiation of green light (λ = 520 nm, 3.7 mW cm^{-2}) for 45 min. Reaction condition: [$OEOMA_{500}$]/[HEBiB]/[$CuBr_2$]/[TPMA] = 300/1/0.75/2.25, [$OEOMA_{500}$] = 360 mM, [DNA or RNA] = 0.1 mg/mL, and [GelGreen or GelRed] = 10x. After polymerization, 50 μL aliquot of the product was taken for ^{1}H NMR analysis without purification to determine monomer conversion. Theoretical molecular weight, $M_{n,NMR}$, was calculated from the conversion assuming quantitative initiation. For GPC analysis (to determine $M_{n,GPC}$ and dispersity), a 200 μL aliquot of the product was mixed with 1 mL of DMF and passed through basic alumina to remove the ATRP catalyst. Absolute molecular weight ($M_{n,abs}$) was determined from $M_{n,GPC}$ using Mark-Houwink calibration following the previously reported procedure.[1, 2]

PET-RAFT polymerization

150 μL of $OEOMA_{500}$ stock, 3 μL of CPADB stock, 2.25 μL of TEOA stock, 25 μL of 10x PBS, 2.5 μL of eosin y stock, and 12.5 μL of salmon DNA stock were combined, thoroughly mixed and the volume was brough to 250 μL by adding water. The reaction mixture was transferred into 250 μL glass insert followed by irradiation of light (λ = 520 nm, 3.7 mW cm^{-2}) for 30 min. Reaction condition: [$OEOMA_{500}$]/[CPADB]/[TEOA]/[eosin y] = 300/1/0.75/0.0125, [$OEOMA_{500}$] = 360 mM, and [salmon DNA] = 0.1 mg/mL. Abbreviations: TEOA (triethanolamine); CPADB (4-cyano-4-(phenylcarbonothioylthio)pentanoic acid).

Photo ATRP using oligonucleotide as the co-catalyst

Synthetic oligonucleotide pellets were dissolved in nuclease free water at the final concentration of 200 μM and utilized as a co-catalyst under the standard photo ATRP condition described above. Briefly, 150 μL of $OEOMA_{500}$ stock, 3 μL of HEBiB stock, 4 μL of $CuBr_2$ stock, 2 μL of TPMA stock, 25 μL of 10X PBS, 2.5 μL of GelGreen stock and oligonucleotide (final concentration of 0.1–0.17 mg/mL) were mixed and the final volume was brought to 250 μL by adding water. The reaction mixture was thoroughly mixed and transferred into a 250 μL glass insert followed by irradiation of light (λ = 520 nm, 3.7 mW cm^{-2}) for 45 min. Reaction condition: [$OEOMA_{500}$]/[HEBiB]/[$CuBr_2$]/[TPMA] = 300/1/0.75/2.25, [$OEOMA_{500}$] = 360 mM, [oligonucleotide] = 0.1–0.17 mg/mL, and [GelGreen] = 10x.

The average molecular weight of deoxyribonucleoside monophosphate (330 g/mole). Therefore, 0.1 mg/mL of nucleic acid is equivalent to 0.3 mM of DNA monomer units. Consequently, for the polymerization using dNTP instead of nucleic acids (Entry 18 in Table 1), 2.06 μL of dNTP mix (10 mM of each) were added instead of oligonucleotides while keeping all other conditions the same.

Analysis of polymerization kinetics

Polymerization kinetics experiments were conducted under standard conditions, with a molar ratio of [$OEOMA_{500}$]/[HEBiB]/[$CuBr_2$]/[TPMA] set to 300/1/0.75/2.25. The initial concentrations were [$OEOMA_{500}$] = 360 mM, 0.1 mg/mL of salmon DNA, and a 10x GelGreen solution.

To prepare the reaction mixture, 2250 μL of $OEOMA_{500}$ stock, 45 μL of HEBiB stock, 60 μL of $CuBr_2$ stock, 30 μL of TPMA stock, 375 μL of 10X PBS, 187.5 μL of salmon DNA stock, 764.9 μL of water, and 37.5 μL of GelGreen stock were combined and thoroughly mixed. Subsequently, 250 μL of the reaction mixture was transferred into a 250 μL glass insert, followed by light irradiation (λ = 520 nm, 3.7 mW cm^{-2}) for a duration ranging from 0 to 120 minutes. After irradiation, the polymerization mixture was removed from the photoreactor. 50 μL of the polymerization products were mixed with D2O for ^{1}H NMR analysis, while the remainder was mixed with DMF for GPC analysis.

Temporal control

The reactions were conducted in 0.5-dram glass vials at 2 mL scale without deoxygenation. The vials were respectively charged with 2 ATRP cocktails with different $CuBr_2$/TPMA concentrations: [$CuBr_2$]/[TPMA] = 0.9 mM/2.7 mM; and [$CuBr_2$]/[TPMA] = 0.3 mM/0.9 mM. Other conditions are the same as standard ATRP condition: [$OEOMA_{500}$] = 360 mM, [HEBiB] = 1.2 mM, 0.1 mg/mL of salmon DNA, 10x GelGreen solution in PBS. Temporal control experiments were performed by turning green LED light (λ = 520 nm, 3.7 mW cm^{-2}) on and off every 30 min and 50 μL of samples were taken at each time point to determine conversion.

Photo ATRP using G-quadruplex forming DNA and Thioflavin T

Prior to use, 120 μL of 0.2 mM 45AG oligonucleotide, 20 μL of 1 M Tris-HCl buffer (pH 7.5), and 20 μL of 1 M KCl were mixed and heated at 90°C for 8 minutes followed by cooling in ice bath for 10 min. For the fluorescence intensity measurement of oligonucleotides stained with Thioflavin T (ThT), 45AG or other oligonucleotides (5 μM) was mixed with ThT (1 μM) in the 50 mM Tris-HCl buffer with 50 mM KCl at the final volume of 150 μL. The fluorescence intensity was recorded in 96-well plate using microplate reader.

Photo ATRP using G-quadruplex forming DNA (45AG) and Thioflavin T was performed as follows. 150 μL of $OEOMA_{500}$ stock, 3 μL of HEBiB stock, 4 μL of $CuBr_2$ stock, 2 μL of TPMA stock, 25 μL of 10X PBS, ThT stock (30–120 μM) and oligonucleotide (0.17 mg/mL) were mixed and the final volume was brought to 250 μL by adding

water. The reaction mixture was thoroughly mixed and transferred into a 250 µL glass insert followed by irradiation of blue light (λ = 450 nm, 5.8 mW cm^{-2}) for 45 min. Reaction condition: [$OEOMA_{500}$]/[HEBiB]/[$CuBr_2$]/[TPMA] = 300/1/0.75/2.25, [$OEOMA_{500}$] = 360 mM, [oligonucleotide] = 0.17 mg/mL, and [ThT] = 30–120 µM.

Photo ATRP using DNA nanoflower as the co-catalyst

DNA nanoflower was synthesized following the previously reported procedures.[3, 4] Briefly, circular DNA template for rolling circle amplification (RCA) was synthesized by temperature annealing of the mixture of primer DNA and 92-mer ssDNA (final concentrations of 2 µM each) and the subsequent addition of T4 DNA ligase (0.03 U/µL) and 10x ligase buffer (30 mM Tris–HCl (pH 7.8), 10 mM $MgCl_2$, 10 mM DTT, and 1 mM ATP). After the overnight incubation at room temperature, RCA was performed through the incubation of the circular DNA with phi29 DNA polymerase (1 U/µL) and phi29 reaction buffer (100 mM Tris-HCl (pH 7.5), 20 mM $MgCl_2$, 20 mM $(NH_4)_2SO_4$, and 8 mM DTT) and dNTP mix (2 mM each) for 20 h at 30°C. Finally, the RCA products underwent sonication and washing via centrifugation. Next, the DNA nanoflower was stained with GelGreen or GelRed (at the final concentration of 200x) for 2 hours. After incubation at room temperature, residual dye in the supernatant was removed via 3 repeats of centrifugations.

For polymerization, 100 µL of DNA nanoflower stock (*c.a.* 0.24 mg/mL of DNA) was dispersed in ATRP cocktail and the volume was adjusted to 600 µL by adding water. The mixture was transferred into the 0.5-dram vial and subsequently deoxygenated by argon purging for 15 min under gentle stirring. Finally, green light (λ = 520 nm, 3.7 mW cm^{-2}) was for 45 min. The reaction mixture was stirred throughout the polymerization to prevent the accumulation of DNA nanoflowers at the bottom. Reaction condition: [$OEOMA_{500}$]/[HEBiB]/[$CuBr_2$]/[TPMA] = 300/1/0.75/2.25, [$OEOMA_{500}$] = 360 mM, and [DNA] = 0.04 mg/mL.

For the SEM analysis, 2 µL of DNFs dispersed in water (30~300 µg/mL) were applied onto a silicon wafer and air-dried for 2 hours at ambient temperature. The samples were then sputter-coated with platinum before SEM imaging and characterized at 5 kV.

Estimation of absolute concentration of NuABDs

Although absolute concentrations for many commercially available dyes are not provided by vendors (usually sold as 10000x concentrate), we have tried to estimate the molarity through alternative means. In our study, the absolute concentration of the dyes was estimated by measuring the mass difference before and after evaporating 80 µL of 10000x dye solutions. The molarity of GelGreen, GelRed and SYBR Gold, was estimated to be 40.3 mM, 32.3 mM, and 18.9 mM, respectively. See Table S9 for the calculation.

Supplementary Figures

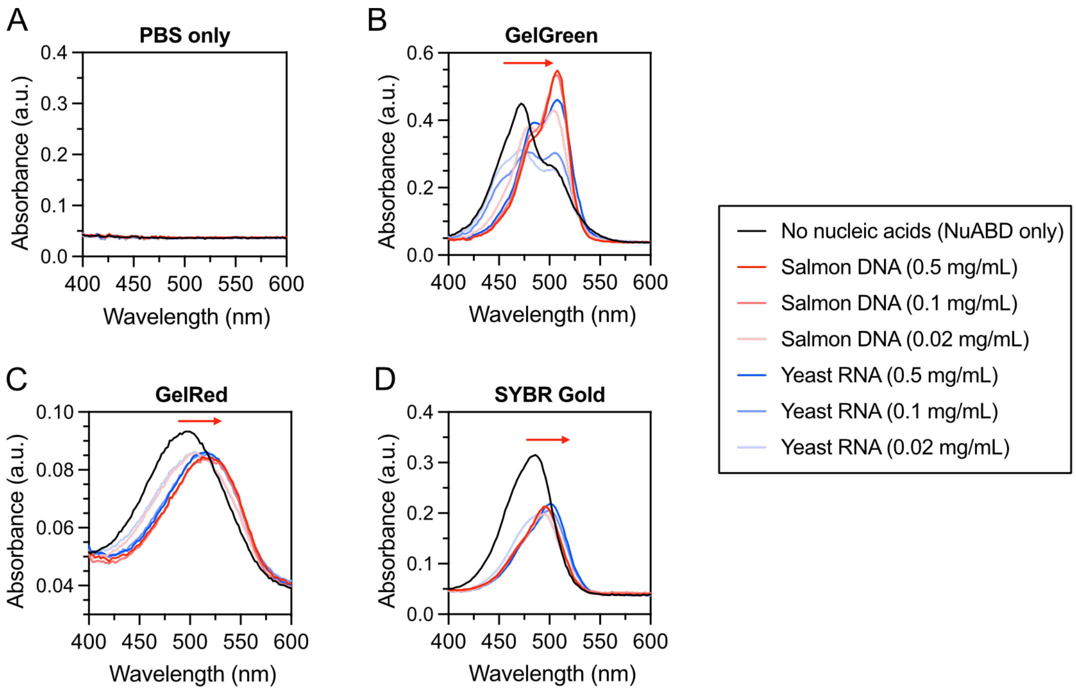

Figure S1. Absorption spectra of (A) PBS; (B) GelGreen; (C) GelRed; and (D) SYBR Gold in PBS, respectively, in the presence of nucleic acids.

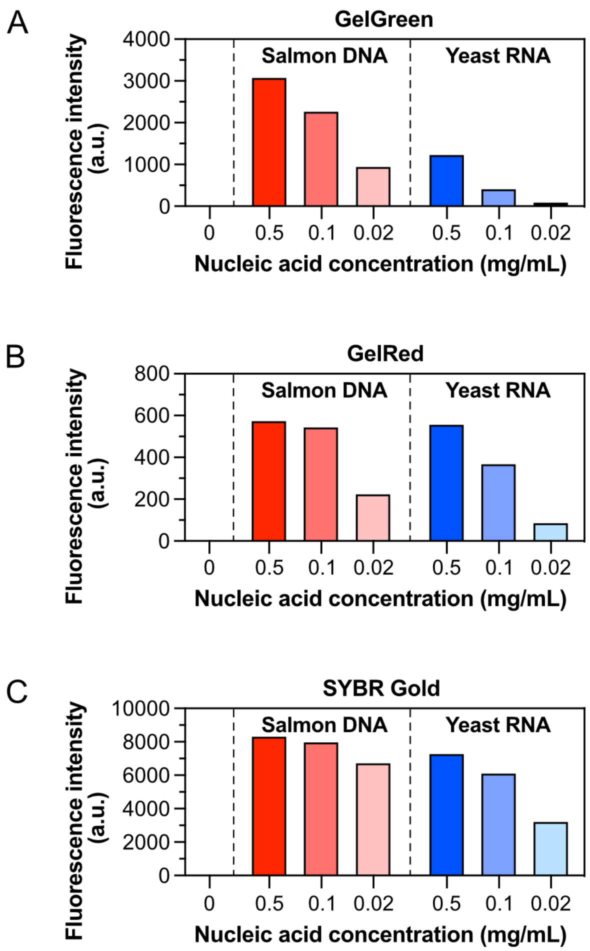

Figure S2. Fluorescence intensity measurement of (A) GelGreen; (B) GelRed; and (C) SYBR Gold in PBS, respectively, in the presence of nucleic acids. Excitation and emission wavelength of each **NuABDs** are as follows: GelGreen (λ_{ex} = 510 nm, λ_{em} = 528 nm), GelRed (λ_{ex} = 526 nm, λ_{em} = 600 nm), and SYBR Gold (λ_{ex} = 495 nm, λ_{em} = 538 nm).

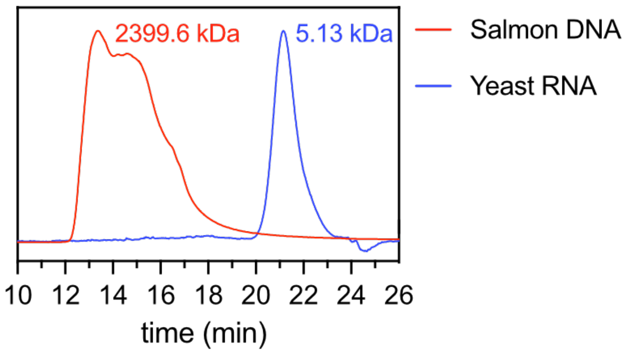

Figure S3. Characterization of biomass DNA (DNA sodium salt from salmon testes) and biomass RNA (from torula yeast) using size exclusion chromatography (SEC) equipped with multi-angle light scattering (MALS) detector. The number-averaged molar mass (M_n) was calculated using a dn/dC value of 0.17 mL/g.

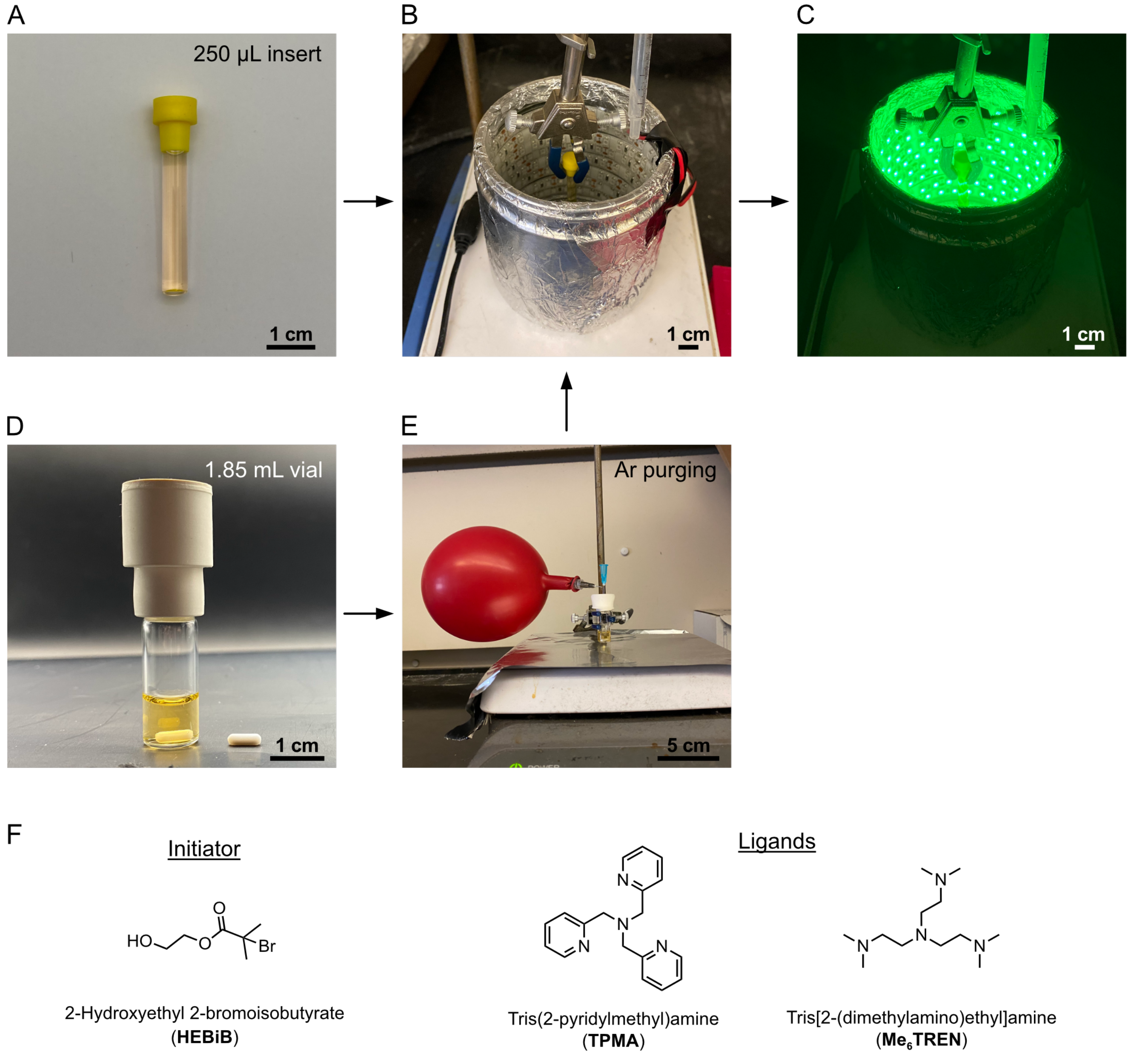

Figure S4. Digital camera images of instruments and polymerization setup. (A) 250 µL of the polymerization cocktail with GelGreen (10x) is placed in a 250 µL flat-bottom glass insert with a cap. (B) A house-made photoreactor. A 250 µL glass insert or a 0.5-dram (1.85 mL) vial was placed inside the glass jar mounted with LED lamps. (C) The photo-polymerization reaction setup with the reaction mixture positioned at the center of the photoreactor. (D) 1.85 mL vial and stir bar for polymerization using DNF as the photocatalyst under continuous stirring. (E) Argon purging setup, using a balloon filled with Argon.

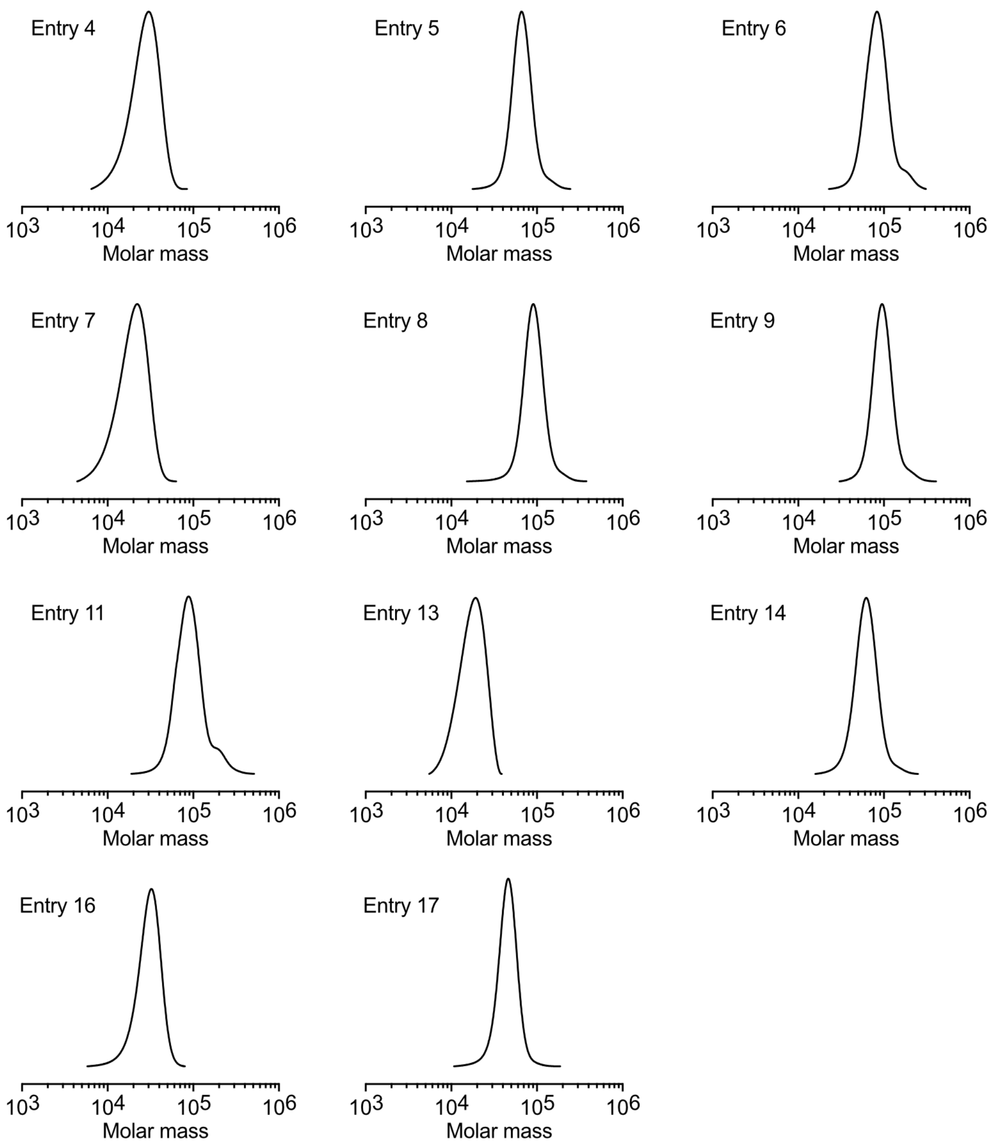

Figure S5. GPC traces for Entries 4–17 in Table 1.

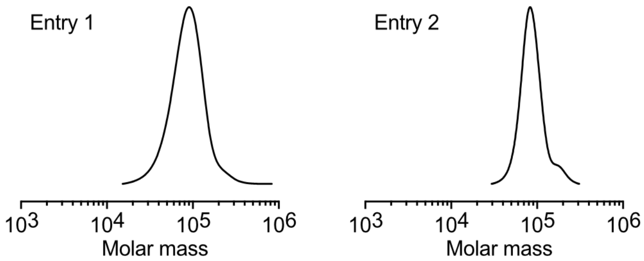

Figure S6. GPC traces for Table S2.

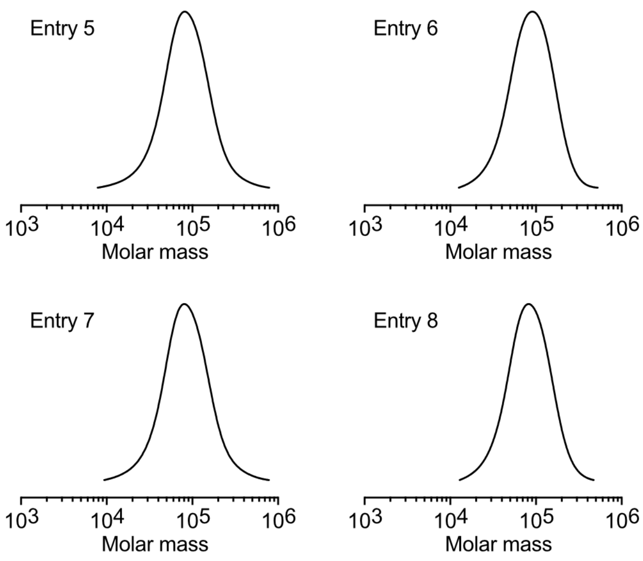

Figure S7. GPC traces for Table S3.

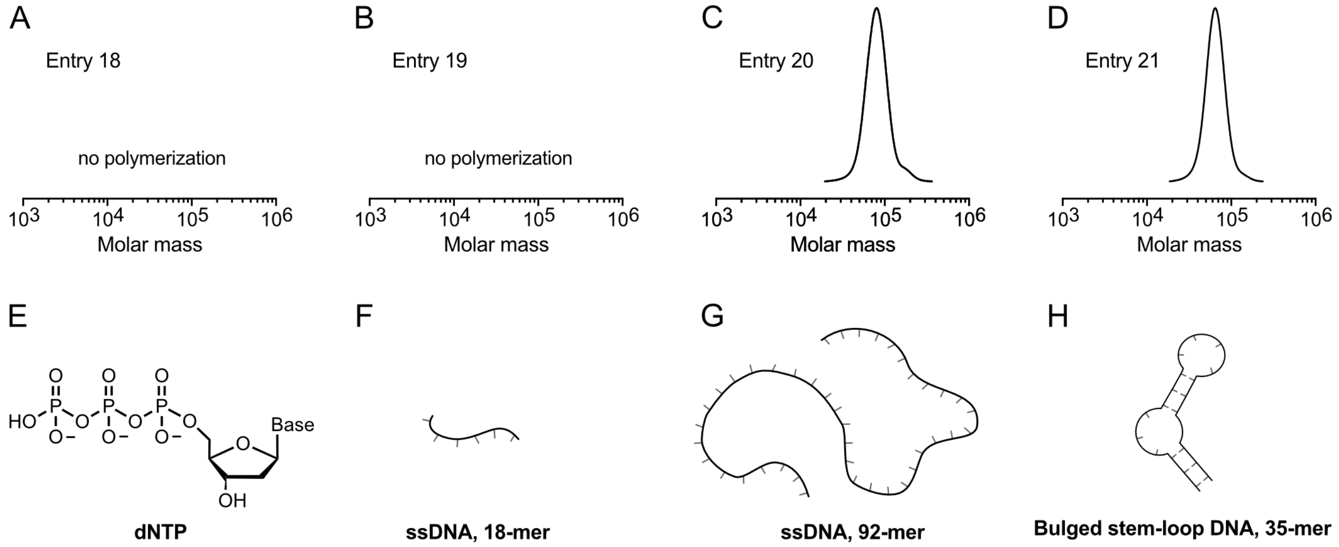

Figure S8. GPC traces for Entries 18–21 in Table 1 with schematic depictions of the oligonucleotide co-catalysts employed in the polymerization processes.

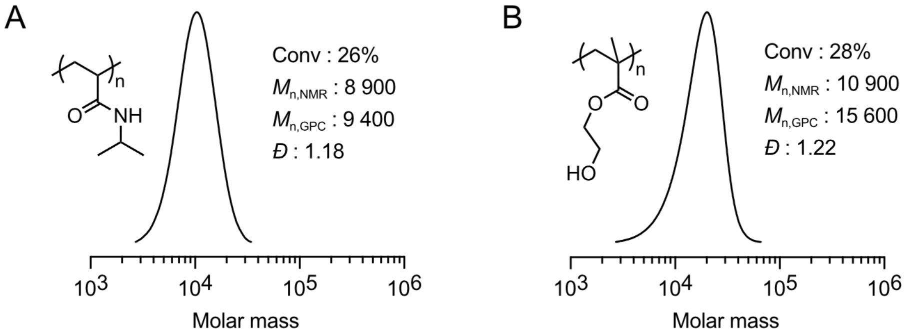

Figure S9. GPC traces of (A) pNIPAM; and (B) pHEMA, synthesized by using Salmon DNA (0.1 mg/mL) and GelGreen (10x). Reaction conditions: [NIPAM or HEMA] = 360 mM, [NIPAM or HEMA]/[HEBiB]/[$CuBr_2$]/[Ligand] = 300/1/0.75/2.25. Me_6TREN and TPMA were used as the ligand for the polymerization of NIPAM and HEMA, respectively. The reactions were performed in PBS under green light irradiation (λ = 520 nm, 3.7 mW cm-2) for 45 min. Monomer conversions were determined by ^{1}H NMR spectroscopy as shown in Figure 1.

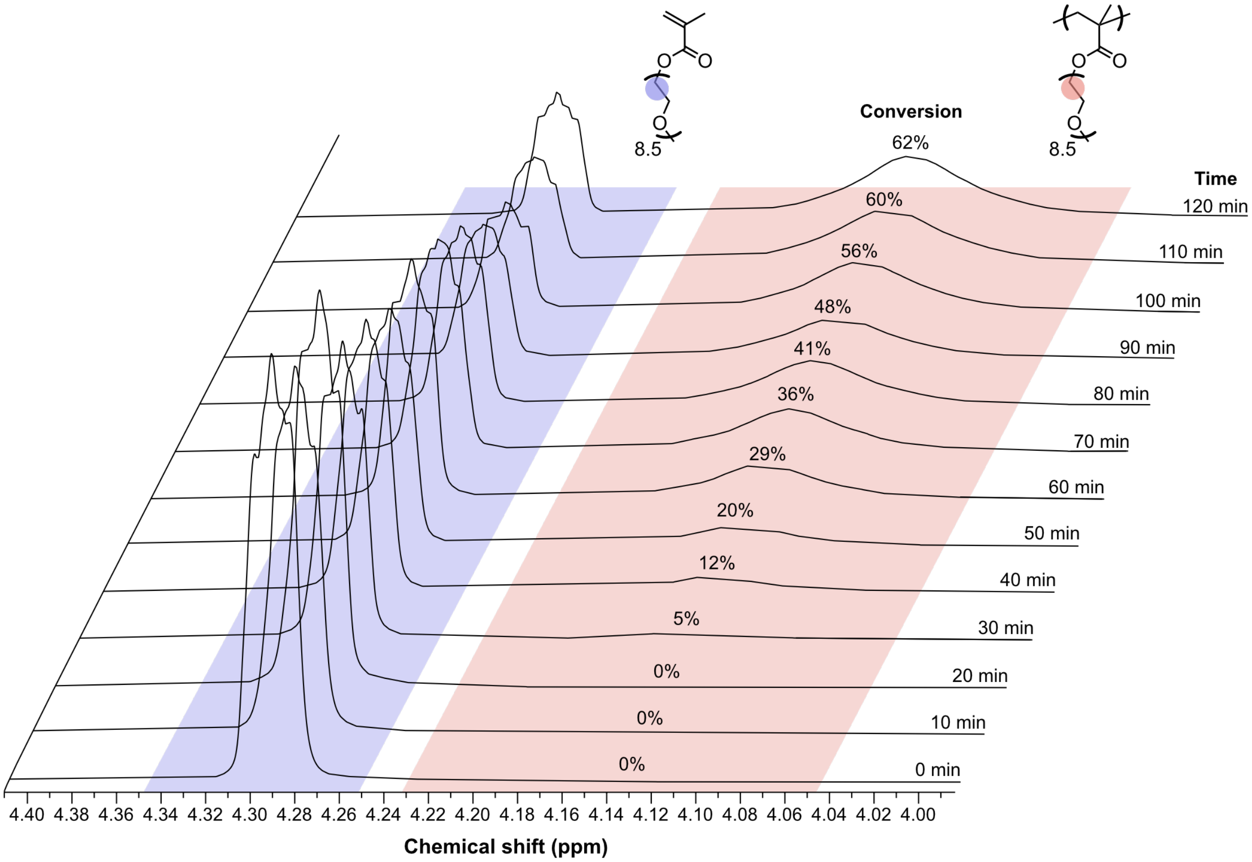

Figure S10. ^{1}H NMR spectra of kinetic analysis shown in Figure 2 and Table S4. Monomer conversion at each time point was determined by comparing the areas under the curves in the blue and red regions, corresponding to the protons in $OEOMA_{500}$ monomer (4.25–4.35 ppm) and the polymerized $OEOMA_{500}$ (4.05–4.23 ppm).

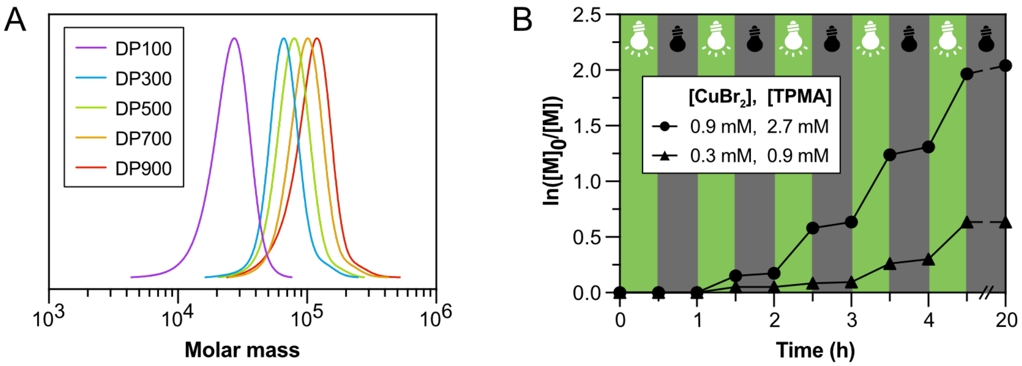

Figure S11. (A) Molecular weight control results. GPC traces of polyOEOMA$_{500}$ were obtained for different target degrees of polymerization within the range of 100 to 900. Polymerization results and the reaction conditions are demonstrated in Table S5. (B) Temporal control of polymerization. Reaction condition: for the reaction of [$CuBr_2$]/[TPMA] = 0.9 mM/2.7 mM, [OEOMA$_{500}$]/[HEBiB]/[$CuBr_2$]/[TPMA] = 300/1/0.75/2.25; for reaction of [$CuBr_2$]/[TPMA] = 0.3 mM/0.9 mM, [OEOMA$_{500}$]/[HEBiB]/[$CuBr_2$]/[TPMA] = 300/1/0.25/0.75. Both reactions were performed in 1x PBS in the presence of salmon DNA (0.1 mg/mL) and GelGreen (10x). ATRP is a radical polymerization method in which unavoidable radical termination occurs between two growing polymer chains. Consequently, Cu^{II}/L cannot revert to its activator form (Cu^{I}/L), resulting in the accumulation of the Cu^{II}/L deactivator. Since low Cu catalysts concentration is used for ATRP, this rapidly consumes activators, eventually leading to polymerization cessation. In the absence of reductive stimuli to (re)generate Cu^{I}/L (e.g., when the light is off), the polymerization remains stopped. Notably, the response of the polymerization system to light can be regulated by adjusting the system's activity. For example, employing active ligands (e.g., TPMA) causes the polymerization to pause almost instantly when the light is off due to the even lower concentration of the ATRP activator during polymerization.[5, 6]

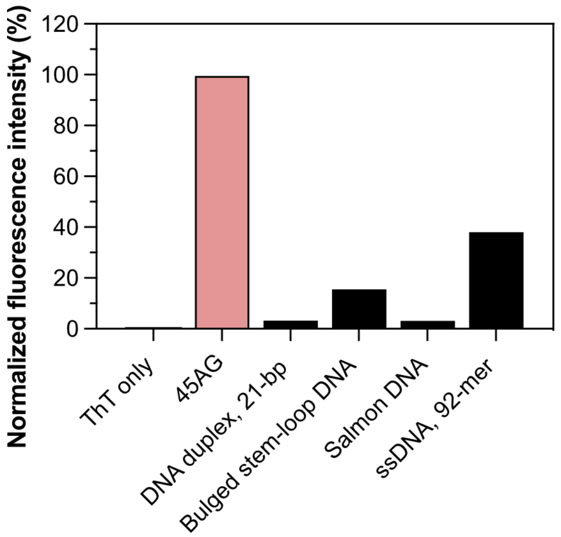

Figure S12. Fluorescence intensity measurement of ThT in the presence of nucleic acids forming secondary structures. Fluorescence intensities were recorded in 96-well plate using microplate reader (λ_{ex} = 446 nm, λ_{em} = 485 nm).

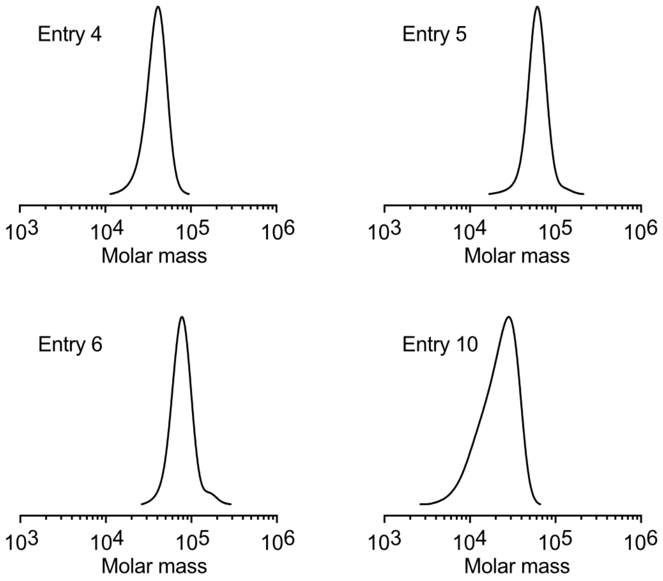

Figure S13. GPC traces for Table S6.

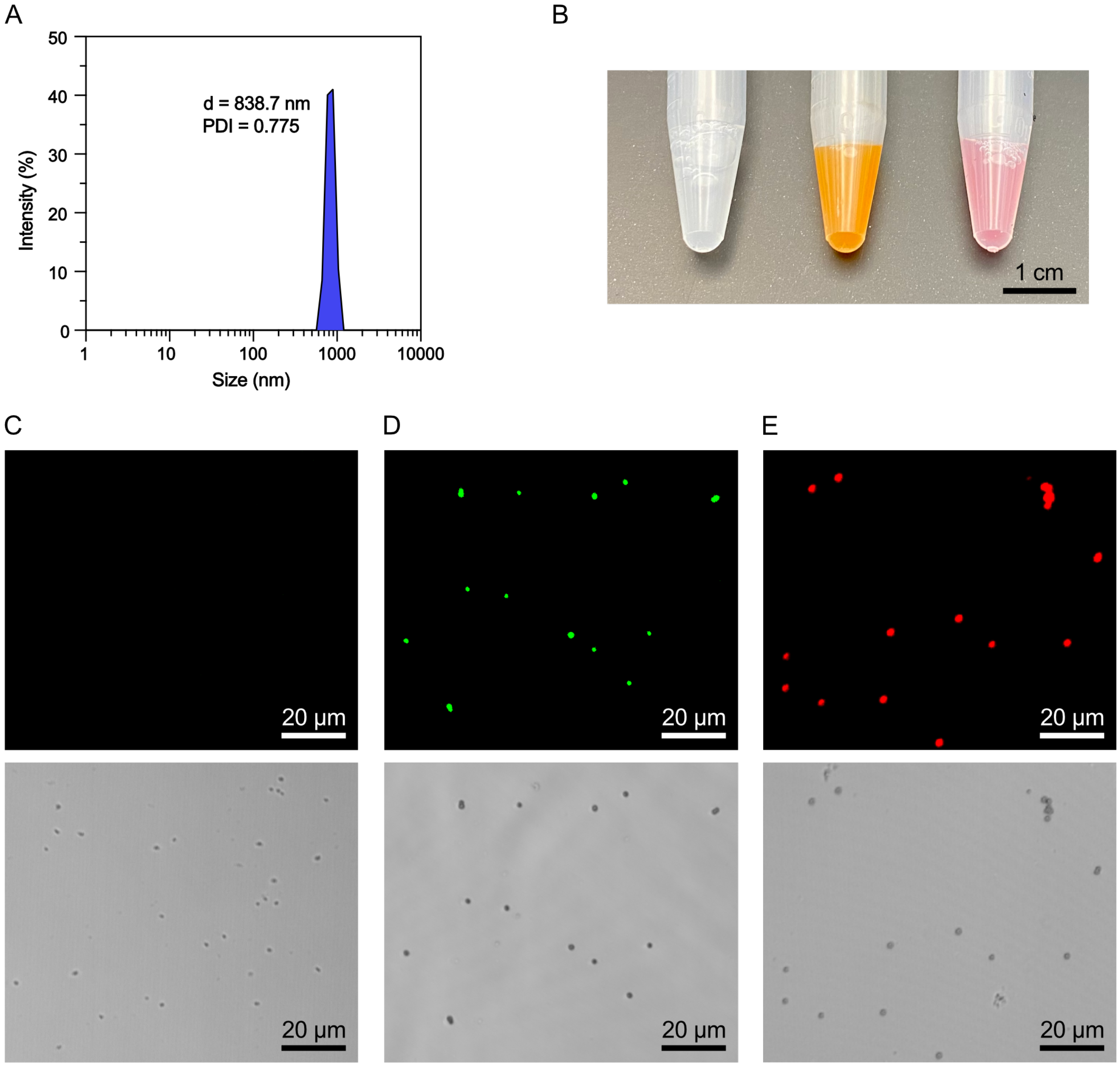

Figure S14. Characterization of DNF. (A) Measurement of DNF particle size using DLS. (B) Digital camera image of DNF dispersed in water. (C–E) (top) Fluorescence microscope images and (bottom) the corresponding brightfield images of (C) unstained DNF; (D) DNF stained with GelGreen; and (E) DNF stained with GelRed, respectively.

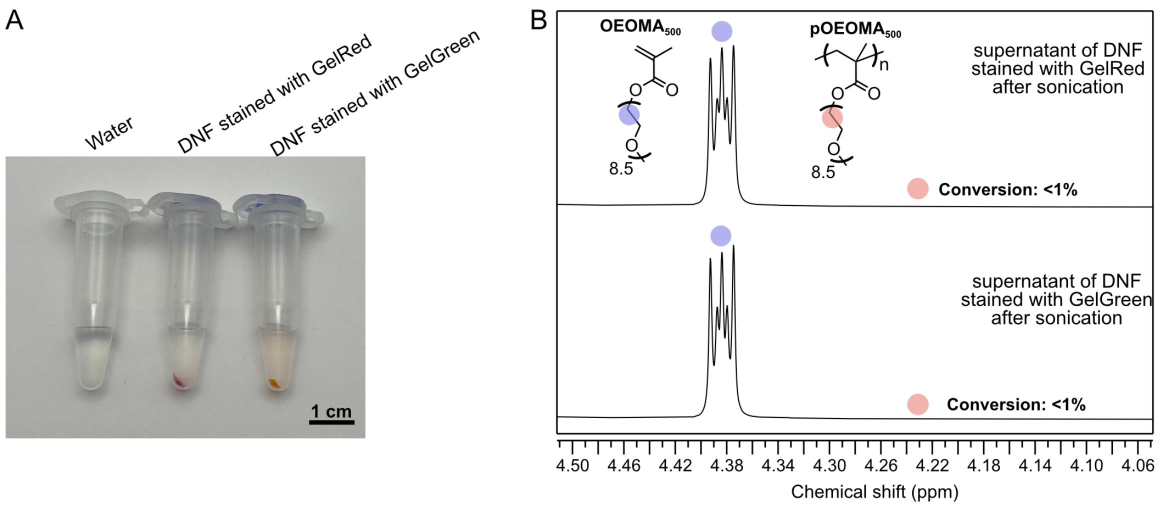

Figure S15. Polymerization using supernatant of centrifuged DNF after 15 min sonication. (A) Digital camera image showing near-transparent color of the supernatant showing negligible leaching of DNA from the DNF after 15 min of sonication (usually <5 min to disperse DNFs). (B) NMR spectra showing negligible polymerization after 45 min of polymerization using the supernatants.

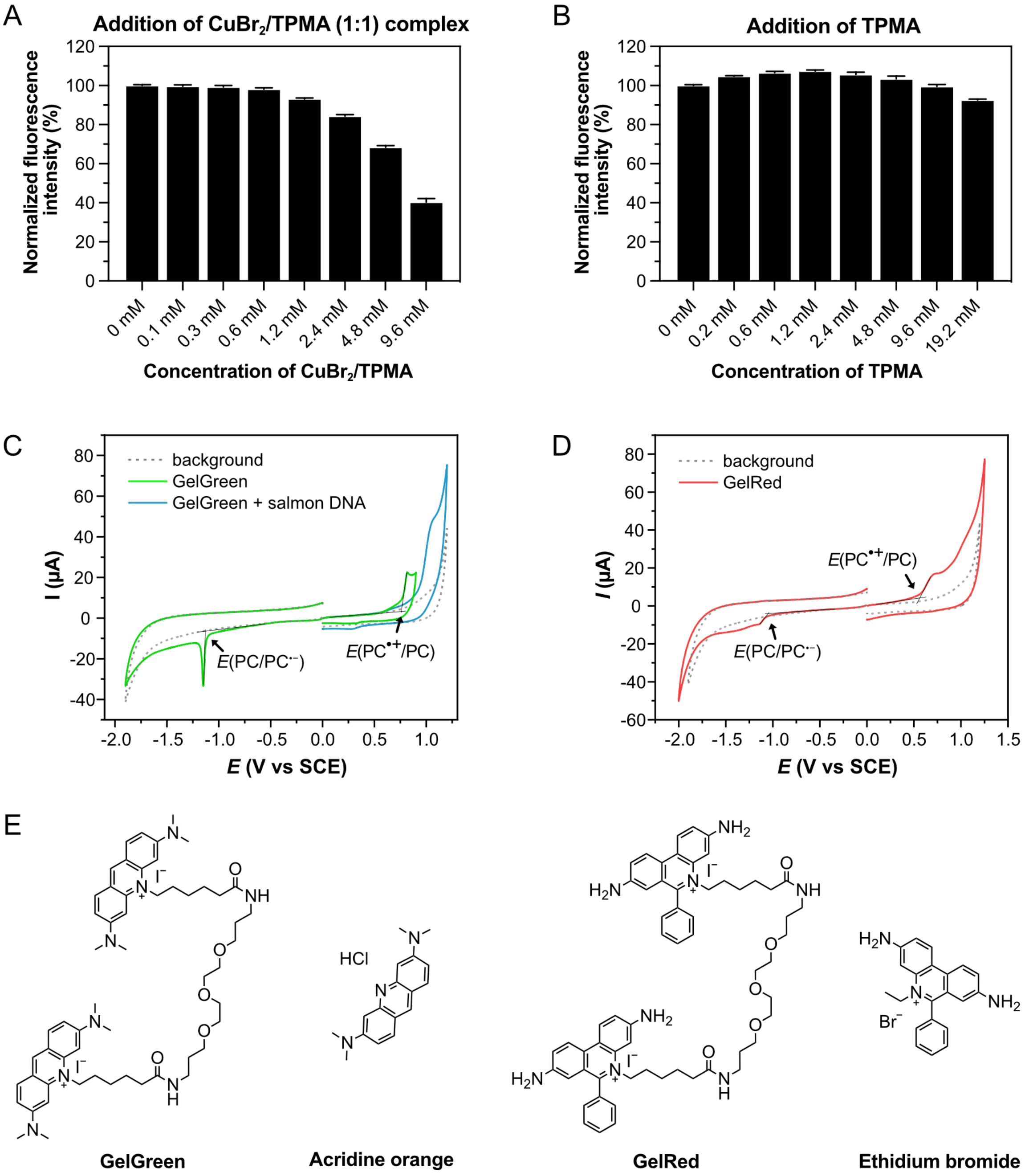

Figure S16. (A, B) Fluorescence intensity of GelGreen (10x) in PBS upon the addition of (A) $CuBr_2$/TPMA (1:1) complex; or (B) TPMA, respectively. Fluorescence intensity was recorded in microplate reader (λ_{ex} = 510 nm, λ_{em} = 528 nm). Standard deviation was calculated from 4 different samples. (C, D) Cyclic voltammetry of **NuABDs** in water + 1x PBS at 0.2 V s^{-1} on a GC electrode (*d* = 0.3 cm). The CVs were recorded in the absence (dotted lines) and in the presence of **NuABDs** (solid lines), including (C) 150X GelGreen; and (D) 150X GelRed. One additional CV was recorded in the presence of both 150X GelGreen and 2 mg/mL Salmon DNA. (E) Chemical structures of homodimeric **NuABDs** (GelGreen and GelRed) and their monomeric units (acridine orange for GelGreen and ethidium bromide for GelRed).

Supplementary Tables

Table S1. Synthetic oligonucleotides used in this study.

Entry	Nucleic acids	Molecular weight	Sequence (5' to 3')
1	18-mer ssDNA	5538.7 g/mole	TGG TAC GTT AGG AAC ATC
2	35-mer bulged stem-loop DNA	10679.0 g/mole	CAA GAT CCC CCG GTG CGA AAA AAC GCA CCA TCT TG
3	92-mer ssDNA	28536.4 g/mole	Phosphate - TCG TTT GAT GTT CCT AAC GTA CCA ACG CAC ACG CAG TAT TAT GGA CTG GTA AAA GCT TTC CGA GGT AGC CTG GAG CAT AGA GGC ATT GGC TG
4	Primer DNA	6730.5 g/mole	TAG GAA CAT CAA ACG ACA GCC A
5	45AG	14290.2 g/mole	G_3 TTA G_3 TTA G_3 TTA G_3 TTA G_3 TTA G_3 TTA G_3 TTA G_3
6	21-bp DNA duplex	12853.4 g/mole	(a) ACA GCT CTG ACT GCT CGA CGT<br>(b) ACG TCG AGC AGT CAG AGC TGT

Table S2. Photo ATRP without excess ligands as an electron donor.[a]

Entry	Salmon DNA	$[CuBr_2]/[TPMA]$	Dye (concentration)	Conv.[b]	$M_{n,NMR}$	$M_{n,GPC}$[c]	$M_{n,Abs}$[d]	$Đ$[c]
1	–	0.9 mM / 2.7 mM	Eosin y (0.015 mM)	77%	96 300	78 100	105 400	1.22
2	0.1 mg/mL	0.9 mM / 2.7 mM	Eosin y (0.015 mM)	84%	126 000	83 700	114 300	1.11
3	0.1 mg/mL	0.9 mM / 0.9 mM	Eosin y (0.015 mM)	3%	4 500	–	–	–
4	0.1 mg/mL	0.9 mM / 0.9 mM	GelGreen (10x)	0%	–	–	–	–
5	0.1 mg/mL	0.9 mM / –	GelGreen (10x)	0%	–	–	–	–

[a] Reaction conditions: $[OEOMA_{500}]$ = 360 mM, $[OEOMA_{500}]/[HEBiB]/[CuBr_2]/[TPMA]$ = 300/1/0.75/0–2.25. The reactions were performed in PBS under the green light irradiation (λ = 520 nm, 3.7 mW cm^{-2}) for 45 min. [b] Monomer conversion was determined by ^1H NMR spectroscopy. [c] Molecular weight and dispersity were determined by using GPC (DMF as eluent) calibrated with PMMA standards. GPC traces of Entries 1 and 2 are shown in Figure S6. [d] Molecular weight was determined by Mark-Houwink calibration following the previously reported procedure.[1, 2]

Table S3. PET-RAFT results.[a]

Entry	Nucleic acid (concentration)	NuABD (concentration)	Conv.[b]	$M_{n,NMR}$	$M_{n,GPC}$[c]	$M_{n,Abs}$[d]	$Đ$[c]
1	–	GelRed or GelGreen (10x)	0%	–	–	–	–
2	Salmon DNA (0.02 mg/mL)	GelRed or GelGreen (10x)	0%	–	–	–	–
3	Salmon DNA (0.1 mg/mL)	GelRed or GelGreen (10x)	0%	–	–	–	–
4	Salmon DNA (0.5 mg/mL)	GelRed or GelGreen (10x)	0%	–	–	–	–
5	–	Eosin y (0.015 mM)	61%	91 500	68 000	88 900	1.49
6	–	Eosin y (0.015 mM) + GelRed (10x)	63%	94 500	73 800	98 200	1.37
7	Salmon DNA (0.1 mg/mL)	Eosin y (0.015 mM)	61%	91 500	69 000	90 500	1.47
8	Salmon DNA (0.1 mg/mL)	Eosin y (0.015 mM) + GelRed (10x)	61%	91 500	70 400	92 700	1.36

[a] Reaction conditions: [$OEOMA_{500}$] = 360 mM, [$OEOMA_{500}$]/[CPADB]/[TEOA] = 300/1/0.75. Abbreviations: TEOA (triethanolamine); CPADB (4-cyano-4-(phenylcarbonothioylthio)pentanoic acid). The reactions were performed in PBS under the green light irradiation (λ = 520 nm, 3.7 mW cm^{-2}) for 45 min. [b] Monomer conversion was determined by ^{1}H NMR spectroscopy. [c] Molecular weight and dispersity were determined by using GPC (DMF as eluent) calibrated with PMMA standards. Corresponding GPC results are shown in Figure S7. [d] Molecular weight was determined by Mark-Houwink calibration following the previously reported procedure.[1, 2]

Table S4. Analysis of polymerization kinetics shown in Figure 2.[a]

Entry	Reaction time	Conv.[b]	$M_{n,NMR}$	$M_{n,GPC}$[c]	$M_{n,Abs}$[d]	$Đ$[c]
1	0 min	0%	–	–	–	–
2	10 min	0%	–	–	–	–
3	20 min	0%	–	–	–	–
4	30 min	5%	7 500	12 700	11 700	1.19
5	40 min	12%	18 000	21 600	22 100	1.18
6	50 min	20%	30 000	30 800	34 100	1.13
7	60 min	29%	43 500	44 200	52 800	1.07
8	70 min	36%	54 000	50 400	61 900	1.09
9	80 min	41%	61 500	53 800	66 900	1.10
10	90 min	48%	72 000	58 000	73 400	1.09
11	100 min	56%	84 000	66 200	86 100	1.09
12	110 min	60%	90 000	69 200	90 800	1.09
13	120 min	62%	93 000	71 700	94 800	1.10

[a] Reaction conditions: [$OEOMA_{500}$] = 360 mM, [$OEOMA_{500}$]/[HEBiB]/[$CuBr_2$]/[TPMA] = 300/1/0.75/2.25. Salmon DNA (0.1 mg/mL) and GelGreen (10x) was used for polymerization. The reactions were performed in PBS under the green light irradiation (λ = 520 nm, 3.7 mW cm^{-2}) for 0–120 min. To maintain a temperature below 37 °C and prevent the photo reactor from overheating during polymerization, a continuous stream of air was introduced into the reactor. [b] Monomer conversion was determined by ^1H NMR spectroscopy. ^1H NMR spectra for the determination of conversion are presented in Figure S10. [c] Molecular weight and dispersity were determined by using GPC (DMF as eluent) calibrated with PMMA standards. [d] Molecular weight was determined by Mark-Houwink calibration following the previously reported procedure.[1, 2]

Table S5. Control over the molecular weights of polymers.[a]

Entry	[M]/[I]/[$CuBr_2$]/[L]	Target DP	Conv.[b]	$M_{n,NMR}$	$M_{n,GPC}$[c]	$M_{n,Abs}$[d]	$Đ$[c]
1	100/1/0.25/0.75	100	48%	24 000	23 400	24 400	1.13
2	300/1/0.75/2.25	300	56%	84 000	63 800	82 300	1.10
3	500/1/1.25/3.75	500	42%	105 000	74 500	99 200	1.11
4	700/1/1.75/5.25	700	37%	129 600	93 000	130 000	1.12
5	900/1/2.25/6.75	900	31%	139 500	101 600	144 600	1.15

[a] Reaction conditions: [$OEOMA_{500}$] = 360 mM, [HEBiB] = 3.6–0.4 mM, [$CuBr_2$] = 0.9 mM, [TPMA] = 2.7 mM. Salmon DNA (0.1 mg/mL) and GelGreen (10x) was used for polymerization. The reactions were performed in PBS under the green light irradiation (λ = 520 nm, 3.7 mW cm^{-2}) for 45 min. To maintain a temperature below 37 °C and prevent the photo reactor from overheating during polymerization, a continuous stream of air was introduced into the reactor. [b] Monomer conversion was determined by ^1H NMR spectroscopy. [c] Molecular weight and dispersity were determined by using GPC (DMF as eluent) calibrated with PMMA standards. Corresponding GPC results are shown in Figure S11A. [d] Molecular weight was determined by Mark-Houwink calibration following the previously reported procedure.[1, 2]

Table S6. Photo ATRP results using G-quadruplex as the co-catalyst shown in Figure 3A–3B.[a]

Entry	Nucleic acid (0.17 mg/mL)	NuABD (concentration)	Conv.[b]	$M_{n,NMR}$	$M_{n,GPC}$[c]	$M_{n,Abs}$[d]	$Đ$[c]
1	–	ThT (30 μM)	0%	–	–	–	–
2	–	ThT (60 μM)	0%	–	–	–	–
3	–	ThT (120 μM)	2%	–	–	–	–
4	45AG	ThT (30 μM)	22%	33 000	37 200	42 800	1.08
5	45AG	ThT (60 μM)	48%	72 000	58 600	74 200	1.08
6	45AG	ThT (120 μM)	73%	109 500	74 500	99 304	1.10
7	DNA duplex, 21-bp	ThT (60 μM)	0%	–	–	–	–
8	Bulged stem-loop DNA	ThT (60 μM)	2%	–	–	–	–
9	Salmon DNA	ThT (60 μM)	1%	–	–	–	–
10	ssDNA, 92-mer	ThT (60 μM)	8%	12 000	18 800	18 700	1.27

[a] Reaction conditions: [$OEOMA_{500}$] = 360 mM and [ThT] = 30–120 μM. [$OEOMA_{500}$]/[HEBiB]/[$CuBr_2$]/[TPMA] = 300/1/0.75/2.25. 0.17 mg/mL of oligonucleotide or Salmon DNA was used for polymerization. The reactions were performed in PBS under the blue light irradiation (λ = 450 nm, 5.8 mW cm^{-2}) for 45 min. [b] Monomer conversion was determined by ^1H NMR spectroscopy. [c] Molecular weight and dispersity were determined by using GPC (DMF as eluent) calibrated with PMMA standards. GPC traces are shown in Figure S13. [d] Molecular weight was determined by Mark-Houwink calibration following the previously reported procedure.[1, 2]

Table S7. Summary of Photo ATRP results using DNFs as the co-catalyst shown in Figure 3C–3E.[a]

Entry	Nucleic acid (0.04 mg/mL)	Conv.[b]	$M_{n,NMR}$	$M_{n,GPC}$[c]	$M_{n,Abs}$[d]	$Đ$[c]
1	DNF, unstained	0%	–	–	–	–
2	DNF + GelGreen	86%	129 000	74 200	98 800	1.15
3	DNF + GelRed	32%	48 000	42 900	50 900	1.08

[a] Reaction conditions: $[OEOMA_{500}]$ = 360 mM, $[OEOMA_{500}]/[HEBiB]/[CuBr_2]/[TPMA]$ = 300/1/0.75/2.25. 0.04 mg/mL of DNFs were used for polymerization. The reactions were performed in PBS the green light irradiation (λ = 520 nm, 3.7 mW cm^{-2}) for 45 min. [b] Monomer conversion was determined by ^1H NMR spectroscopy. [c] Molecular weight and dispersity were determined by using GPC (DMF as eluent) calibrated with PMMA standards. [d] Molecular weight was determined by Mark-Houwink calibration following the previously reported procedure.[1, 2] Prior to polymerization, the reaction mixtures (600 μL) were purged with Argon for 15 min under gentle stirring.

Table S8. Electrochemical and photochemical properties of the photocatalysts. All potentials in V vs SCE, and energies in eV.

NuABD	$E(PC/PC^{•-})$	$E(PC^{•+}/PC)$	E_{00}	$E(PC^{*}/PC^{•-})$	$E(PC^{•+}/PC^{*})$	τ_{PC}	$\tau_{PC\text{-}DNA}$
GelGreen	-1.13[a]	0.76	2.33[b]	1.20	-1.57	1.7 ns (0.25)[c]	5.5 ns (0.7)[c]
GelRed	-1.05	0.56	2.06[d]	1.01	-1.50	1.6 ns (0.02)[e]	22.1 ns (0.40)[e]

[a] $E(PC/PC^{•-})$ = -1.18 V vs SCE was reported for acridine orange.[7] [b] The transition energy was estimated from the maximum of the emission wavelength, λ_{max} = 531 nm, as $E_{00} = hc/\lambda_{max}$, where *c* is the speed of light in vacuum and *h* is Planck's constant. [c] Data for acridine orange. Quantum yield in parenthesis.[8] [d] The transition energy was estimated from the maximum of the emission wavelength, λ_{max} = 602 nm, as $E_{00} = hc/\lambda_{max}$, where *c* is the speed of light in vacuum and *h* is Planck's constant. [e] For ethidium bromide. Quantum yield in parenthesis.[9] Electrochemical and photochemical properties of the photocatalysts. All potentials in V vs SCE, and energies in eV.

Table S9. Estimation of molarity of commercially available 10000x NuABD stocks. 80 μL of 10000x NuABD were taken for the estimation.

	A	B	C	D = C–B	E = D/A*1000	F = E/80*1000
NuABD	Molar mass	Vial only	Vial + NuABD, after evaporation	mass	μmoles in 80 μL	molarity
GelGreen	1198.4	4922.6 mg	4926.5 mg	3.9 mg	3.25	40.7 mM
GelRed	1239.1	4646.4 mg	4649.4 mg	3.1 mg	2.50	31.3 mM
SYBR Gold	495.7	4952.8 mg	4953.6 mg	0.8 mg	1.61	20.1 mM

Table S10. Estimation of GelRed and SYBR Gold bound to salmon DNA. C_b and C_T represent the bound dye concentration and total dye concentration, respectively.

Entry[a]	Nucleic acid (conc.)	NuABD (conc.)	Conv.	C_b	C_b/C_T
1	–	GelRed (10x)	0%	0 μM	0%
2	Salmon DNA (0.1 mg/mL)	GelRed (10x)	0%	0 μM	0%
3	Salmon DNA (0.02 mg/mL)	GelRed (10x)	8%	7.5 μM	24%
4	Salmon DNA (0.1 mg/mL)	GelRed (10x)	49%	31.0 μM	99%
5	Salmon DNA (0.5 mg/mL)	GelRed (10x)	55%	31.3 μM	100%
6	–	SYBR Gold (20x)	0%	0 μM	0%
7	Salmon DNA (0.1 mg/mL)	SYBR Gold (10x)	6%	20.0 μM	100%
8	Salmon DNA (0.1 mg/mL)	SYBR Gold (20x)	50%	40.0 μM	100%

[a] Entries 1–8 corresponds to the Entries 1, 2, 7, 8, 9, 12, 13, and 14 in Table 1, respectively.

Supplementary Discussions

Cyclic voltammetry analysis of NuABDs

GelGreen is composed of dimeric linked acridine orange dyes, while GelRed is composed of dimeric linked ethidium bromide (Figure S16E). In the literature, both acridine orange and ethidium bromide show irreversible cyclic voltammetry,[10] where the electron transfer is accompanied by successive proton transfer events. Similarly, both GelGreen and GelRed show irreversible oxidation and reduction waves in 1x PBS in water (Figure S16C and S16D). The reduction of GelGreen shows a sharp symmetric peak (at onset potential $E_{on,red}$ = -1.13 V vs SCE) suggesting that the catalyst is partially adsorbed on the surface of the electrode. Due to the irreversible cyclic voltammetry waves observed for both dyes, their standard potentials for reduction and oxidation were estimated from their onset potentials, $E_{on,red}$ and $E_{on,ox}$, which were obtained from the intersection of two tangents drawn at the rising reduction (or oxidation) current and the baseline current of the CV curves. The values of reduction and oxidation potentials are summarized in Table S8.

The addition of DNA significantly alters the CV response of GelGreen, because of the intercalation of the dye. The original oxidation peak of GelGreen (at $E_{on,ox}$ = +0.76 V vs SCE) in Figure S16C disappears, and a new peak emerges at a more positive potential (new $E_{on,ox}$ = +0.92 V vs SCE). This new peak is associated to the direct oxidation of DNA. It is noteworthy that the intercalated GelGreen is not electrochemically active, as observed for other intercalating dyes.[11] This lack of electrochemical activity stems from the redox center (i.e., the dyes) being embedded within a large macromolecule (i.e., nucleic acid scaffolds), which does not produce an electrochemical response on a flat electrode.

The potentials for the photocatalysts in the excited state were estimated from the excitation energy of the photocatalysts (E_{00}), according to the following equations:

$$E^{o}_{PC*/PC^{\bullet-}} = E^{o}_{PC/PC^{\bullet-}} + E_{00} \quad \text{(equation S1)}$$

$$E^{o}_{PC^{\bullet+}/PC*} = E^{o}_{PC^{\bullet+}/PC} - E_{00} \quad \text{(equation S2)}$$

The results are summarized in Table S8.

It should be noted that both singlet and triplet excited states can contribute to the photoinduced polymerization. Some long-lived triplet states have been reported for acridine orange and ethidium bromide.[12] These triplet states, however, are formed with very low quantum efficiency (10^{-3}). Our reasoning focuses on the redox potentials and on

the increased lifetime of singlet states, which have been thoroughly characterized in the literature, but a contribution from long lived triplet states (although formed with low quantum yield) is possible.

Effect of DNA binding on the photophysical properties of the NuABDs

Binding with DNA has a significant impact on the lifetime and quantum yield of emission, as evidenced by the data collected in Table S8 for the bare photocatalysts (PC) and the PC-DNA complexes. However, binding to DNA induces only a slight change in the energy of the ground and excited states. Indeed, upon the addition of DNA, there is only a minor alteration in the shape and maxima of the absorption and emission spectra of the photocatalysts (see Figure S1).[9] Unfortunately, as explained in the previous section, direct electrochemical information cannot be obtained from the PC-DNA complex due to its lack of electrochemical activity. In conclusion, at first approximation we can estimate that the potential and energy level of the dyes are similar before and after complexation with DNA, and that the main effect of binding to DNA is an increase in emission lifetime and yield (Table S8).

Additional mechanistic discussions

In this system, energy transfer mechanism is unlikely due to the limited overlap between the emission band of the dye and the photocatalytically active band of Cu catalysts. Cu catalysts typically require excitation within their UV absorption band to induce a photocatalytic effect (i.e., to initiate polymerization), with a maximum wavelength of 400 nm. In contrast, the energy of the excited-state dye is considerably lower (emission wavelength > 500 nm). Therefore, energy transfer pathway is negligible in our system.

Also, redox potentials were calculated for $CuCl/CuCl_2$-based system. Polymerization in the aqueous phase is often performed in PBS which contains ca. 140 mM of chloride anion. Meanwhile, the concentration of $CuBr_2$ is in the range of 0.3 to 0.9 mM. As a consequence, the bromine in the $Br\text{-}Cu^{II}/L$ is rapidly replaced to chloride and most copper will be in the form of $Cl\text{-}Cu^{II}/L$. Therefore, the electrochemical study for ATRP in aqueous phase utilized an electrolyte that is composed of chloride anions employing Cu-Cl system.

Quantification of GelRed bound to DNA

The fraction of GelRed and SYBR Gold bound to salmon DNA was calculated as C_b/C_T, where C_b and C_T represent the bound dye concentration and total dye concentration, respectively. The calculation was performed based on the McGhee-von Hippel model of ligand-substrate binding (equation S3).[13] For GelRed, the necessary parameters were obtained from the article by Rocha et al.[14] In equation S3, K_i is intrinsic equilibrium association constant (K_i = 1.8×10^7 M^{-1}), r is C_b/C_{bp} (C_{bp} is the concentration of base-pair = 30, 150, or 750 μM), C_f is C_T–C_T, and n is an exclusion number of 3.7 which is the number of DNA base pairs occupied by an intercalated dye. A similar calculation was carried out for the SYBR Gold, using the appropriate literature coefficients.[15]

As shown in Table S10, C_b/C_T correlates well with monomer conversion implying that bound dye predominantly facilitates polymerization due to its enhanced photophysical properties. No calculation was carried out for GelGreen, since there are no available literature values.

$$\frac{r}{C_f} = K_i(1-nr)\left[\frac{1-nr}{1-(n-1)r}\right]^{n-1} \qquad \text{(equation S3)}$$

References

1. Szczepaniak, G.; Jeong, J.; Kapil, K.; Dadashi-Silab, S.; Yerneni, S. S.; Ratajczyk, P.; Lathwal, S.; Schild, D. J.; Das, S. R.; Matyjaszewski, K., Open-air green-light-driven ATRP enabled by dual photoredox/copper catalysis. *Chem. Sci.* **2022,** *13* (39), 11540-11550.
2. Jeong, J.; Szczepaniak, G.; Das, S. R.; Matyjaszewski, K., Expanding the architectural horizon of nucleic-acid-polymer biohybrids by site-controlled incorporation of ATRP initiators in DNA and RNA. *Chem* **2023,** *9* (11), 3319-3334.
3. Lee, J. B.; Peng, S.; Yang, D.; Roh, Y. H.; Funabashi, H.; Park, N.; Rice, E. J.; Chen, L.; Long, R.; Wu, M., A mechanical metamaterial made from a DNA hydrogel. *Nat. Nanotechnol.* **2012,** *7* (12), 816-820.
4. Lv, Y.; Hu, R.; Zhu, G.; Zhang, X.; Mei, L.; Liu, Q.; Qiu, L.; Wu, C.; Tan, W., Preparation and biomedical applications of programmable and multifunctional DNA nanoflowers. *Nat. Protoc.* **2015,** *10* (10), 1508-1524.
5. Dadashi-Silab, S.; Lee, I.-H.; Anastasaki, A.; Lorandi, F.; Narupai, B.; Dolinski, N. D.; Allegrezza, M. L.; Fantin, M.; Konkolewicz, D.; Hawker, C. J., Investigating temporal control in photoinduced atom transfer radical polymerization. *Macromolecules* **2020,** *53* (13), 5280-5288.
6. Rolland, M.; Truong, N. P.; Whitfield, R.; Anastasaki, A., Tailoring polymer dispersity in photoinduced iron-catalyzed ATRP. *ACS Macro Lett.* **2020,** *9* (4), 459-463.
7. Romero, N. A.; Nicewicz, D. A., Organic Photoredox Catalysis. *Chemical Reviews* **2016,** *116* (17), 10075-10166.
8. Kubota, Y.; Steiner, R. F., Fluorescence decay and quantum yield characteristics of acridine orange and proflavine bound to DNA. *Biophysical Chemistry* **1977,** *6* (3), 279-289.
9. Chib, R.; Raut, S.; Sabnis, S.; Singhal, P.; Gryczynski, Z.; Gryczynski, I., Associated anisotropy decays of ethidium bromide interacting with DNA. *Methods and Applications in Fluorescence* **2014,** *2* (1), 015003.
10. Hu, X.; Wang, Q.; He, P.; Fang, Y., Spectroelectrochemistry study on the electrochemical reduction of ethidium bromide. *Anal. Sci.* **2002,** *18* (6), 645-650.
11. Minasyan, S.; Tavadyan, L.; Antonyan, A.; Davtyan, H.; Parsadanyan, M.; Vardevanyan, P., Differential pulse voltammetric studies of ethidium bromide binding to DNA. *Bioelectrochemistry* **2006,** *68* (1), 48-55.
12. Olmsted III, J.; Kearns, D. R., Mechanism of ethidium bromide fluorescence enhancement on binding to nucleic acids. *Biochemistry* **1977,** *16* (16), 3647-3654.
13. McGhee, J. D.; von Hippel, P. H., Theoretical aspects of DNA-protein interactions: co-operative and non-co-operative binding of large ligands to a one-dimensional homogeneous lattice. *J. Mol. Biol.* **1974,** *86* (2), 469-489.
14. Crisafuli, F.; Ramos, E.; Rocha, M., Characterizing the interaction between DNA and GelRed fluorescent stain. *European Biophysics Journal* **2015,** *44*, 1-7.
15. Kolbeck, P. J.; Vanderlinden, W.; Gemmecker, G.; Gebhardt, C.; Lehmann, M.; Lak, A.; Nicolaus, T.; Cordes, T.; Lipfert, J., Molecular structure, DNA binding mode, photophysical properties and recommendations for use of SYBR Gold. *Nucleic Acids Res.* **2021,** *49* (9), 5143-5158.
